# Miniature coiled artificial muscle for wireless soft medical devices

**DOI:** 10.1126/sciadv.abm5616

**Published:** 2022-03-11

**Authors:** Mingtong Li, Yichao Tang, Ren Hao Soon, Bin Dong, Wenqi Hu, Metin Sitti

**Affiliations:** 1 Physical Intelligence Department, Max Planck Institute for Intelligent Systems, 70569 Stuttgart, Germany.; 2 Institute of Functional Nano & Soft Materials (FUNSOM), Jiangsu Key Laboratory for Carbon-Based Functional Materials & Devices, Soochow University, Suzhou, Jiangsu 215123, P. R. China.; 3 School of Mechanical Engineering, Tongji University, Shanghai 201804, P. R. China.; 4 Institute for Biomedical Engineering, ETH Zürich, 8092 Zürich, Switzerland.; 5 School of Medicine and College of Engineering, Koç University, 34450 Istanbul, Turkey.

## Abstract

Wireless small-scale soft-bodied devices are capable of precise operation inside confined internal spaces, enabling various minimally invasive medical applications. However, such potential is constrained by the small output force and low work capacity of the current miniature soft actuators. To address this challenge, we report a small-scale soft actuator that harnesses the synergetic interactions between the coiled artificial muscle and radio frequency–magnetic heating. This wirelessly controlled actuator exhibits a large output force (~3.1 N) and high work capacity (3.5 J/g). Combining this actuator with different mechanical designs, its tensile and torsional behaviors can be engineered into different functional devices, such as a suture device, a pair of scissors, a driller, and a clamper. In addition, by assuming a spatially varying magnetization profile, a multilinked coiled muscle can have both magnetic field–induced bending and high contractile force. Such an approach could be used in various future untethered miniature medical devices.

## Teaser

Miniature wireless magnetic coiled muscle design enables the large force output required by biomedical applications.

## INTRODUCTION

Wireless small-scale soft-bodied medical devices that are several millimeters or smaller can safely and adaptively navigate through confined spaces, such as those inside the human body (*1*). This unique capability can potentially be disruptive in diverse medical applications (*2*), such as minimally invasive surgery (*3*) and local and on-demand therapeutic operations (*4*, *5*). Various optical (*6*, *7*), thermal (*8*), and acoustic (*9*) soft actuation methods have been explored for such untethered miniature soft-bodied devices (*10*). While the softness does endow these actuators with the capability to have large programmed deformations and safe interaction with the environment, it also limits their output force and weight-normalized work capacity (mentioned as “work capacity” hereafter). Typically, soft actuators exhibit small force output and large deformation. The material softness makes it hard to store and release large amounts of mechanical energy; hence, they cannot be used in device and robot applications requiring large force output and high work capacity. For example, the output force of the existing magnetic soft actuators were calculated to saturate at around 60 μN (see “Calculation of the force and work capacity of the magnetic soft actuator” section; figs. S1 and S2), irrespective of increasing the external magnetic field amplitude. On the contrary, some medical procedures, such as pinching, clamping, and cutting (*11*, *12*), require devices with much higher force output (> 1 N), which is ~10^5^ times higher than the maximum capacity of the previously reported magnetic soft actuators.

At the centimeter and larger length scales, coiled artificial muscles have been promising actuators that can be fabricated by continuously twisting polymer fibers into a coiled shape (*13*, *14*). Recent works have proposed that the coiled muscles can have higher work capacity and larger output force than several popular actuation approaches (*15*, *16*). Specifically, the coiled muscles can have 50 times larger work capacity than the biological skeletal muscles and can deliver force output more than 1000 times of their own weight when composed of carbon nanotubes, shape memory polymers, and fishing lines (*16*–*18*). In addition, their output force and the work capacity can be greatly enhanced by tailoring the component or the structure of the precursor fiber of the muscle, such as adding the graphene oxide (GO) platelets inside to the precursor fiber or designing a tough sheath on its surface (*17*, *19*), which are poised to advance the fields of humanoid robots, prosthetic limbs, and microfluidic devices.

Here, we integrate coiled artificial muscles into wireless miniature soft medical devices to extend their medical applications to the ones that require an integrated tool with a large output force and high work capacity. We propose coiled artificial muscles that are composed of a trilayer structure: muscle core, active sheath, and protective sheath. Each layer has a specific function. The muscle core is used to form the coiled structure of the fiber in the continuous twisting process. In the critical active sheath, the embedded GO and superparamagnetic Fe_3_O_4_ nanoparticles increase the sheath toughness and enable radio frequency (RF)–magnetic field–based wireless heating, respectively. The function of the protective sheath is to mechanically and thermally reduce the direct exposure of the inner layers to the external environment. Such trilayer design, together with the introduction of the magnetic nanoparticles and GO, contributes to the untethered actuation with a large force output (~3.1 N) and high work capacity (~3.5 kJ/kg). By integrating this muscle to different medical devices, various wireless miniature medical devices, such as a suture device, scissor, and driller, that require high force output and work capacity are possible. Furthermore, actuation strain, output force, and work capacity of the muscle can be further amplified by integrating with an energy-storing bistable structure (*20*). To demonstrate this, a miniature clamper with a 14 N output force is designed for tissue pinching and wound clamping. In addition, the coiled muscle can be coated with an extra magnetization sheath with neodymium-iron-boron (NdFeB) and poly(dimethylsiloxane) (PDMS) soft composite. Thus, magnetization profile can be encoded in a multilinked coiled muscle to enable magnetic deformation and high output contractile force.

Indeed, previous work has used the microwave to wirelessly drive coiled muscles without sheath structure (*14*). To enhance the output performance of the coiled muscle, Mu *et al.* (*19*) have used hot air to drive sheath structures. In comparison, the coiled muscles reported here combine wireless actuation with sheath structures to improve its performance. In addition, the Fe_3_O_4_ nanoparticles used can introduce RF magnetic heating more likely being used in biomedical applications (*21*) than the microwave. In comparison with the conventional magnetic soft actuator (*1*, *22*, *23*), the coiled muscle adds tensile and torsional motion to the conventional magnetic soft actuator, enriching its design space and application fields. It has a 2 × 10^5^-fold increase in force output and a 1.75 × 10^4^-fold increase in work capacity (Table 1). By using this proposed coiled muscle, we have proposed five miniature device prototypes to make good use of this coiled muscle for potential practicality. Such coiled muscle actuator can potentially be used in a variety of untethered miniature soft medical device and robot applications, expanding the current capabilities of the previously proposed wireless miniature soft medical devices and robots (*4*, *23*–*25*).

**Table 1.** Comparison of different types of soft and muscle actuators in terms of actuation strain, work capacity, and energy density. LCE, liquid crystal elastomer; HASEL, hydraulically amplified self-healing electrostatic actuator; IPMC, ionic polymer-metal composites.

**Type**	**Actuation strain (%)**	**Work capacity (kJ/kg)**	**Energy density (kJ/m^3^)**	**Ref.**
Coiled artificial muscle	~30.6	~3.5	~2000	This work
Skeletal muscle	~30	~0.07	~20	(*28*)
McKibben actuator	~38	~1.1	~0.28	(*29*)
LCE	~30	~0.03	~30	(*30*)
Bio-hybrid	~15	~0.001	~1	(*31*)
HASEL	~60	~0.07	~0.23	(*32*)
IPMC	~3	~0.001	~3	(*33*)
Magnetic soft robot	~50	~2 × 10^−4^	~0.15	“Calculation of the force and work capacity of the magnetic soft actuator” section

## RESULTS

### Actuator design, fabrication, and mechanism

**Fig. 1.**
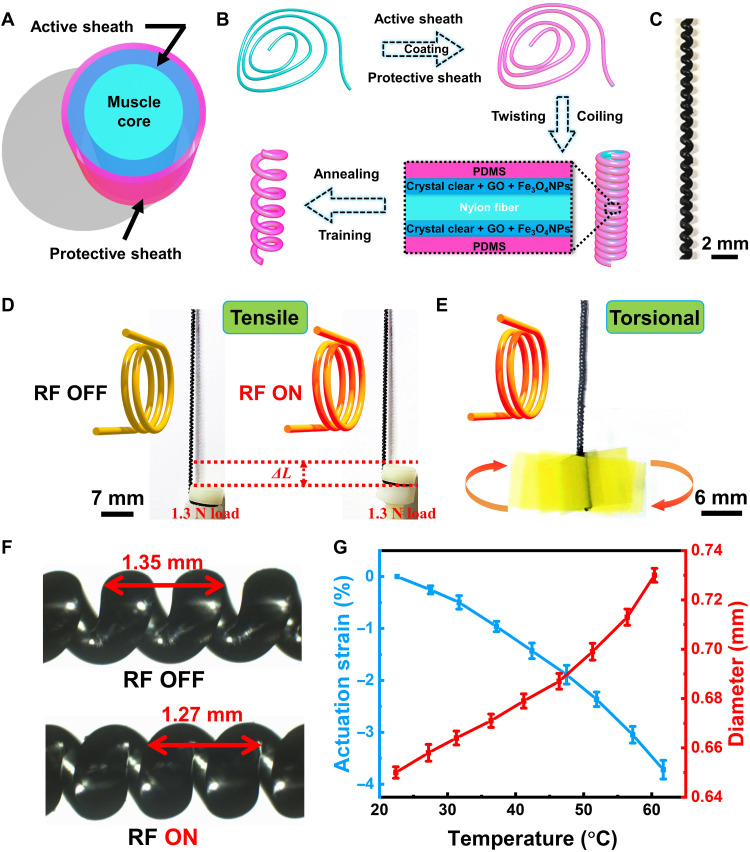
Design, fabrication process, actuation modes, and actuation mechanism of the proposed magnetically heated coiled muscle. (**A**) The schematic picture of the coiled muscle for the cross-sectional structure before coiled. (**B**) The fabrication process of the coiled muscle includes coating, twisting and coiling, annealing, and training process. (**C**) Photo of the fabricated 1-mm-diameter coiled muscle. (**D**) The contractile actuation and (**E**) torsional actuation modes of the coiled muscle under RF magnetic heating. (**F**) Photos of the structural changes of the coiled muscle before and after RF magnetic heating. (**G**) The length and diameter changes of the trilayer precursor fiber before coiling with different temperature conditions. The images in (D) and the overlaid image in (E) and (F) are obtained from movies S1, S2, and S4, respectively.

The design of the proposed muscle actuator is shown in Fig. 1A. It consisted of three layers: a high-strength nylon fiber as the muscle core that provides the restoring stiffness (*19*), a protective sheath at the outermost surface to insulate the inner layers both mechanically and thermally to the external environment (see Materials and Methods and fig. S3), and an active layer between the aforementioned two. The active layer was composed of three elements: Crystal Clear matrix (Crystal Clear is a rigid urethane casting resin, which is a heat-cured commercial product), superparamagnetic Fe_3_O_4_ nanoparticles, and mechanical strength–enhancing GO platelets. The trimorph fiber was lastly twisted to form the coiled muscle.

The actuator was fabricated through a customized droplet-coating technique (Fig. 1B) (*26*). As the first step, a 0.45-mm-diameter nylon fiber was dipped into the uncured Crystal Clear resin solution, which is mixed with Fe_3_O_4_ nanoparticles and GO platelets, to create the active sheath. Next, it was coated with PDMS using a similar droplet-coating process to form the protective sheath. After the PDMS layer was fully cured, its cross-sectional structure was characterized by scanning electron microscopy (SEM), as shown in fig. S4A. The thickness of each sheath can be controlled by repeating the droplet-coating method multiple times. Then, this final coiled actuator was obtained by continuously twisting the above trimorph fiber to form the coiled structure first and then annealing and training it. As shown in the optical microscopy image (Fig. 1C) and the zoomed SEM image (fig. S4B), the resulting actuator has a coiled shape, which is approximately 1 mm in diameter.

**Fig. 2.**
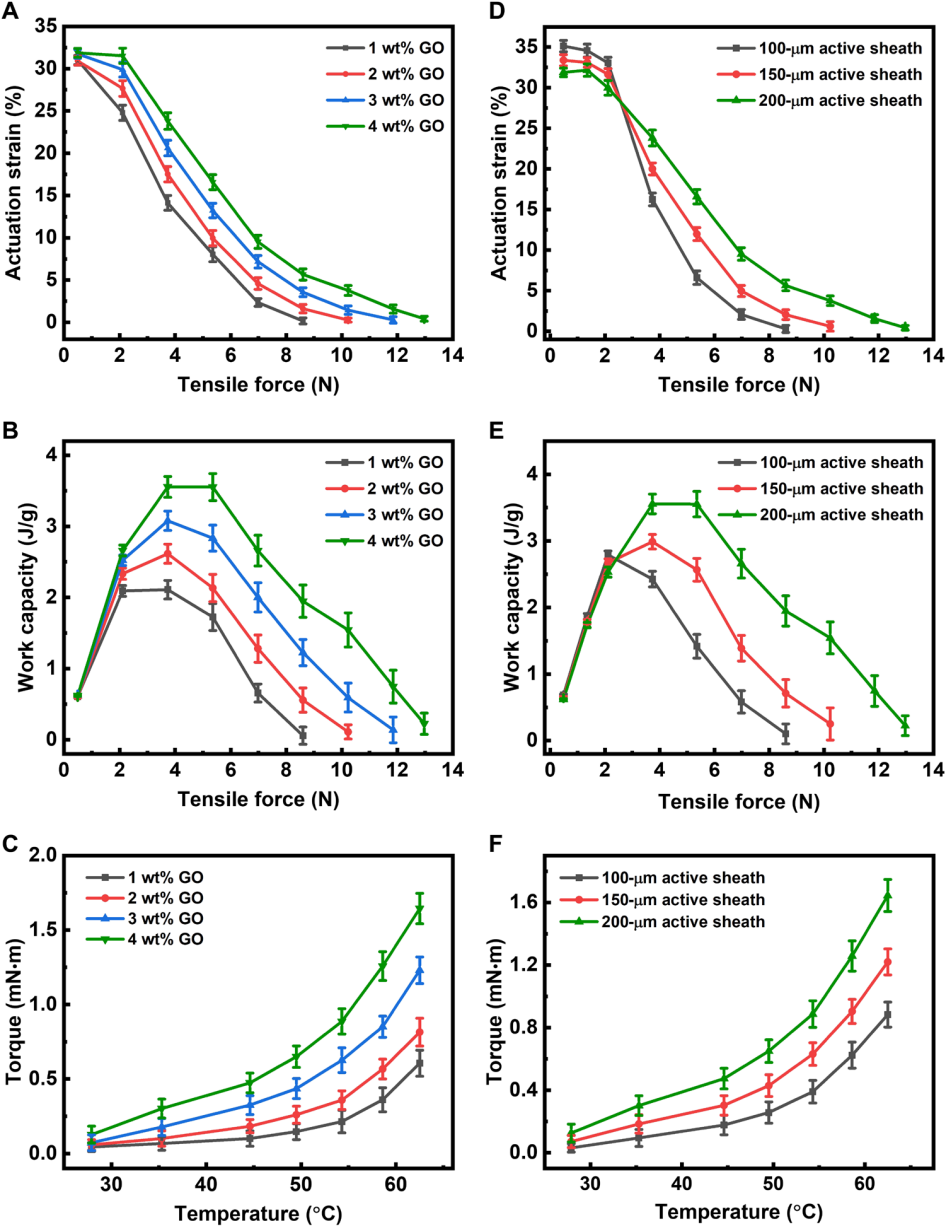
Characterization of the coiled muscle performance. The influence of (**A** and **B**) the GO platelet concentration and (**D** and **E**) the active sheath thickness on the muscle actuation strain and work capacity under different tensile forces, respectively. The torque output of the coiled muscle with different (**C**) GO platelet concentrations and (**F**) active sheath thicknesses.

The coiled muscle was wirelessly activated by heating the embedded magnetic nanoparticles through an external RF magnetic field generator. The coiled muscle had two operation modes under wireless RF-magnetic heating. First, when both ends are allowed to move axially but constrained from rotation, it exhibited contractile actuation. As shown in Fig. 1D and movie S1, it could pull a 1.3 N target with a strain of 30%. It could even pull up to 12.6 N with a reduced strain (1%), which is discussed later in Fig. 2A. As shown in fig. S5, the tensile force output of the coiled muscle with a constant strain of 0.1% could reach ~3.1 N. Second, when its one end was fixed and it was free to rotate, it exhibited torsional actuation, as shown in Fig. 1E and movie S2, where its torque could reach ~1.76 mN·m. In addition, we characterize the change of actuation strain and torsional stroke in this contractile and torsional actuation, respectively (fig. S6). It indicates that the contractile is a reversible actuation, while torsional is a one-time actuation. This is due to the fact that there is no constrain during the torsional actuation, which releases the strain energy generated in the twisting and coiling fabrication process. To reverse the torsional actuation, we can design coiled muscles in two sections with different twisted directions. The design is shown in fig. S7. By actuating the lower section, the overall actuator rotates counterclockwise, opposite to its clockwise design. By actuating the upper section, the actuator rotates clockwise (movie S3).

The proposed coiled muscles demonstrate characteristics that are inaccessible to previous twisting muscles, including magnetic responsiveness, enhanced mechanical output, and better compatibility with biological tissues, toward wireless medical device applications. Its actuation mechanism is due to the untwisting process of the fiber caused by thermal contraction of the fiber in the longitudinal direction and thermal expansion in the radial direction. We studied the mechanism by recording the actuation process under an optical microscope. As shown in Fig. 1F and movie S4, there was a contraction between the coil pitch from 1.95 to 1.77 mm before and after RF magnetic heating. We also characterized the length and the diameter of the trimorph structure fiber before the twisting and coiling process under different temperatures. As indicated in Fig. 1G, there was a contraction in the axial direction of 3.7% and an expansion in the radial direction of around 11.3%, further verifying the actuation mechanism.

The key feature of this trimorph design lies in the composition of the active sheath (the intermediate layer), which includes the resin matrix, magnetic nanoparticles, and GO platelets. First, Crystal Clear resin was used as the matrix because of its intrinsically high toughness and large expansion ratio (see Materials and Methods). Both were beneficial in delivering high stroke and high mechanical output. Second, Fe_3_O_4_ nanoparticles enabled the magneto-thermal effect, making it possible to wirelessly actuate the muscle by an RF-magnetic field (note that Fe_3_O_4_ nanoparticles themselves also played a role in toughening the muscle) (*27*). Third, GO platelets, because of their unique two-dimensional (2D) geometry, substantially aided the fiber strain energy storage, especially in the case of being twisted, much more than that of nanoparticles or carbon nanotubes as toughening agents (*17*). Here, we introduced the GO platelets into the active layer (which is located near the outermost surface) because the expansion force was more effective when they were acting near the margin of the yarn (fig. S8), where the strain energy was the maximum (*16*, *17*).

### Performance characterization

The performance of the magnetically heated coiled muscle, such as its actuation strain and work capacity and torque output, was highly dependent on the GO platelet concentration and the active sheath thickness. These two factors were systematically characterized in Fig. 2. First, Fig. 2 (A to C) shows the results of increasing the platelet concentration from 1 to 4 weight % (wt%) while fixing the active sheath thickness to 200 μm; 4 wt%–concentrated coiled muscle delivered a higher actuation strain. The load-optimized work capacity and torque increased from 2.46 J/g and 0.66 mN·m to 3.5 J/g and 1.76 mN·m by tuning the toughening agent from 1 to 4 wt%, respectively. This is because the GO platelets exhibited substantial bending and twisting behavior inside the fiber, which improved the stored mechanical energy during twisting and coiling (*17*).

Second, Fig. 2 (D to F) shows the result of increasing the sheath thickness from 100 to 200 μm while fixing the GO platelet concentration to 4 wt%. Figure 2D shows the result of the actuation strain. When the load was less than 2.1 N, the coiled muscle with a thicker sheath thickness had a lower actuation strain. This was because of the intercoil contact (*18*). When the load was higher than 2.1 N, the coiled muscle with a thicker sheath thickness had a higher actuation strain. Figure 2 (E and F) shows that the load-optimized work capacity and torque could respectively increase from 2.19 J/g and 1.01 mN·m to 3.5 J/g and 1.76 mN·m by increasing the sheath thickness from 100 to 200 μm. Such a result agrees with the previous literature findings, where thickening the outer layer, rather than the core, is much more effective in improving the artificial muscle performance (*19*). Note that the active sheath tended to break in our experiments when the platelet concentration and the active sheath thickness exceeded 4 wt% and 200 μm, respectively. Therefore, no further measurements were conducted beyond these parameters. In addition, we have characterized the coiled muscle in deionized water. It includes surface temperature, tensile actuation strain, tensile work capacity, and torsional torque, shown in fig. S9. By comparing with Fig. 2, the output performance of the coiled muscle inside the water is similar to that in the air. This could be attributed to the insulation of the protective sheath.

Last, we further compared the mechanical performance of the proposed coiled muscles with other types of soft actuators, as summarized in the “Calculation of the force and work capacity of the magnetic soft actuator” section, fig. S10A, and Table 1. The current coiled muscle has a higher work capacity than popular soft actuators, including the skeletal muscle (*28*), McKibben actuator (*29*), liquid crystal elastomer (*30*), bio-hybrid actuator (*31*), hydraulically amplified self-healing electrostatic actuator (*32*), ionic polymer-metal composites (*33*), and magnetic soft actuators (see “Calculation of the force and work capacity of the magnetic soft actuator” section). In the category of magnetic soft actuators, these reported magnetically heated coiled muscles exhibit ~2 × 10^5^ times higher actuation force output than the previous magnetic soft actuators and ~1.75 × 10^4^ times higher work capacity, greatly improving the mechanical performance of the magnetic soft actuators and expanding their capabilities to medical device applications requiring high force and work density output.

### Application demonstrations

**Fig. 3.**
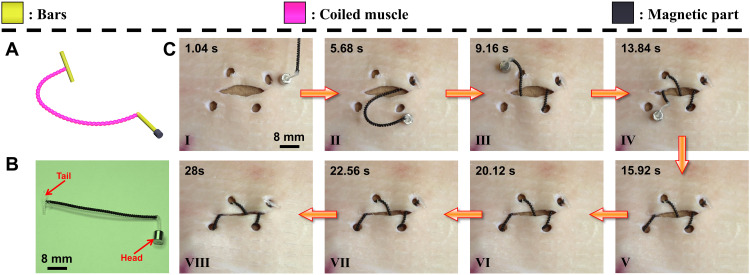
A wireless suturing device demonstration. (**A**) Schematic showing the design of the suturing device. (**B**) The suturing device consists of two printed obstacle panels, a coiled muscle, and a cylindrical NdFeB magnet. (**C**) The wrapping motion of the suturing device is controlled by the external magnetic field gradient–based pulling forces generated by an external permanent magnet and the wound suturing process under RF magnetic heating.

Using the given magnetically heated coiled muscle prototype, we report its several proof-of-concept demonstrations toward wireless medical device applications (fig. S10B). The first demonstration shows a suture device (Fig. 3). Suturing is a critical aspect of most surgeries (*34*–*36*). Conventional suturing relies on articulated tools and physical manipulation of the suturing device. Because of the large footprints of the manipulation tools, conventional suturing usually takes large surgical invasiveness, which will cause more tissue damage, longer recovery times, or other associated infections (*37*). Here, we demonstrate a wireless suture medical device performing wound suturing on a pigskin ex vivo. It was possible due to the precise magnetic field control and large output force of the coiled muscle. As shown in Fig. 3 (A and B), the suturing device was composed of two bars on the two sides of the coiled muscle and a magnetic part, which was a cylindrical NdFeB magnet, at the forefront. The bars were made by the pure nylon fiber used to prevent the rotation of the coiled muscle under RF-magnetic heating. It also acted as an anchor during the contraction of the coiled muscle to enclose the wound. The front NdFeB magnet was used to pull and guide the coiled muscle in wrapping around the wound by the gradient pulling of an external permanent magnet. Note that the Fe_3_O_4_ nanoparticles used in the active sheath were too weak to generate enough pulling force through this magnet field generated by a permanent magnet. The whole suturing process is shown in Fig. 3C and movie S5, which was divided into two steps. In the first step, the suturing device was wrapped around the wound under the magnetic field (I to V in Fig. 3C), which demonstrated the flexibility (small bending stiffness) of the coiled muscle. Note that we made holes on the pigskin beforehand to facilitate easier piercing. Because of the high stiffness of the porcine skin, currently, it is not possible to penetrate the tissue even though we added a suture needle on one end of the actuator. However, this penetration could be achieved by using a sharper needle or a greater magnetic gradient force. In the second step, the RF-magnetic heating was turned on to contract the artificial muscle for wound closure (VI to VIII in Fig. 3C), indicating the large output force of the coiled muscle. We did a control experiment by using an Instron tensile measurement device to pull the head of the device to close the wound. It required a force of 1.21 N with 29.6% actuation strain to realize the same effect (fig. S11). Therefore, this demonstration indicates that the proposed coiled muscle exhibits both high flexibility and large output force. When RF-magnetic heating was turned off, the recovery of the contracted muscle could reopen the wound. However, we observed little reopening. This could be due to the friction between the pigskin and the substrate where the pigskin was attached to. This wound reopening issue can be solved by making a proper knot (*38*) or by designing an anchoring structure, such as a bistable structure (*20*), at the end of the coiled muscle in the future. In addition, the device is intended to remain in the body until the wound recovers. Currently, the outermost protective layer of the coiled muscle actuator is composed of PDMS, which is a biocompatible material (*39*). However, further biocompatibility has to be tested to justify the overall biocompatibility of the device. Last, how to release it after the wound is healed is worthy of further investigation.

**Fig. 4.**
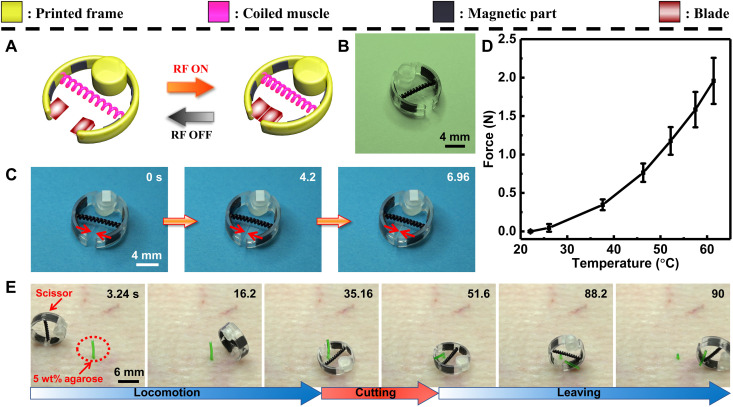
A wireless medical scissor device demonstration. (**A**) Schematic showing the cutting behavior of the designed scissor device. (**B**) The image of the scissor device consists of a 3D-printed polymer frame, coiled muscle actuator, magnetic part (NdFeB microparticles embedded inside an Ecoflex-30 silicone rubber matrix), and two cutting blades. (**C**) Cutting behavior of the scissor device under RF magnetic heating. (**D**) The cutting force of the scissor device was measured during the cutting process. (**E**) Video snapshots of the device rolling on the surface by external magnetic field rotation using a permanent magnet to reach a target agarose gel post and cutting the post by RF heating.

In the second demonstration shown in Fig. 4, a pair of scissors medical device was designed. This scissor function is surgically important for cutting the epidermis or internal biological tissues (*40*). Tetherless scissors can potentially reach confined in vivo sites and implement the above functions (*41*). As shown in Fig. 4 (A and B), we designed the scissor medical device consisting of a circular printed frame, magnetic parts [as a composite of NdFeB microparticles embedded inside a silicone elastomer (Ecoflex 00-30) matrix], and two glass blades assembled with the coiled muscle. The circular shape and NdFeB magnetic composite parts were used to enable the rolling locomotion of the device on solid tissues under the control of an external permanent magnet, which is teleoperated. The blades were carefully designed not to impede the rolling motion and hurt the surrounding tissue during the locomotion. Under RF-magnetic heating, this scissor device exhibited cutting behavior based on the contraction of the coiled muscle (Fig. 4C and movie S6). We quantitatively characterized the cutting force of this scissor device as shown in Fig. 4D. The force increased with the heating temperature, and the maximal force was ~2 N. In Fig. 4E, we demonstrated the feasibility of the scissor device by cutting a synthetic gel pillar toward cutting tissues in future medical applications. After being rolled to the workplace with a manually rotating magnetic field by using an external NdFeB permanent magnet (locomotion part in Fig. 4E), the device is carefully controlled to orient its blades on a gel pillar made of 5 wt% agarose (a synthetic tissue-like material) (*42*). Next, RF-magnetic heating was turned on to activate the artificial muscle and eventually cut the pillar in half (cutting part in Fig. 4E). After cutting, the scissor device could be driven away from the agarose tissue under similar magnetic field control with the locomotion part (leaving the scene part in Fig. 4E). The above demonstration is also shown in movie S7.

**Fig. 5.**
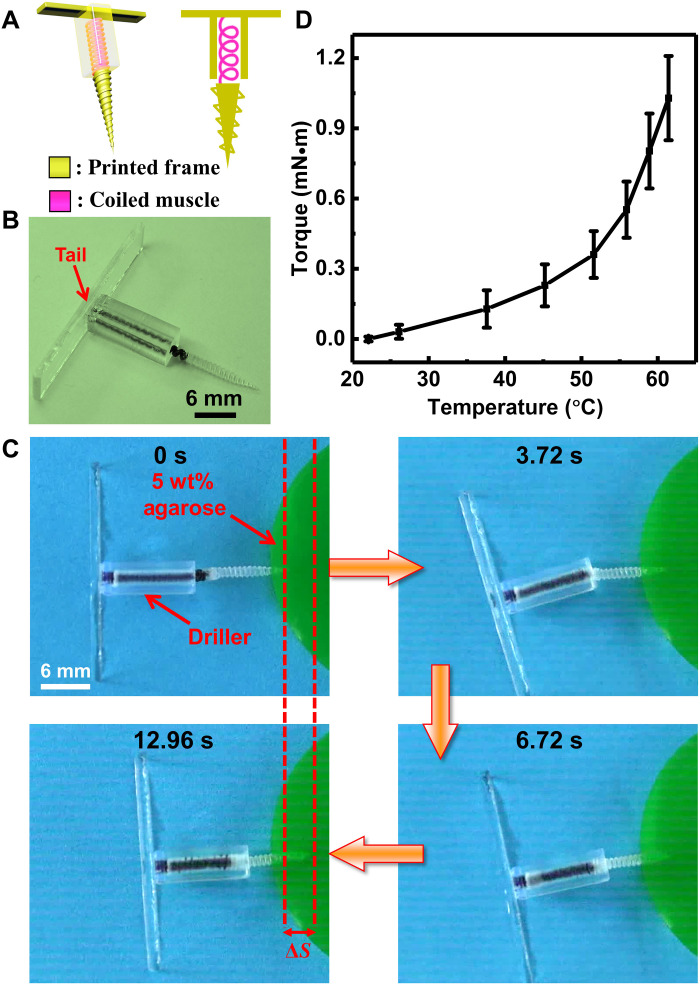
A wireless medical driller device demonstration. (**A**) Schematic of the designed driller device. (**B**) The image of the driller device consisting of the 3D-printed polymer frame and the coiled muscle. (**C**) Video snapshots of the device drilling an agarose gel surface, resembling a soft biological tissue, for a Δ*S* distance, when magnetically heated. (**D**) The measured drilling torque of the device under RF heating.

Third, a wireless medical driller device using torsional actuation of the coiled muscle under RF-magnetic heating was demonstrated (Fig. 5). Wireless drillers are promising for medical applications including navigation (*43*), clot removal (*44*), or tissue biopsy (*45*) as the treatment procedure is untethered, precise, and minimally invasive. However, one challenge of these wireless drillers is to achieve large output torques. For example, the output torque of a previously reported magnetic driller was only 149 μN·m (*46*). Our designed driller device adopted a previously unidentified mechanism that leveraged the high energy-releasing capability of the coiled muscle during the untwisting process to achieve large torque output to overcome these challenges. As can be seen in Fig. 5 (A and B), the coiled muscle was assembled with a screw at the bottom end and a chassis with a rectangular tail. The rectangular tail of the chassis was tethered on one side of the coiled muscle, which was used to limit the rotation of one end of the muscle during the drilling process. The device could drill into the 5 wt% agarose gel under RF-magnetic heating (Fig. 5B and movie S8) as a synthetic tissue-like material for drilling demonstration. The drilling displacement was about 3 mm, indicated as Δ*S* in Fig. 5C, and the drilling torque could reach ~1.2 mN∙m (Fig. 5D). Currently, the device needs to rest on something to function, as the tail chassis needs to counteract the torque being applied by the screw drilling. The reaction force can be distributed in a wider area by designing a larger tail, with a consideration of the maximal pressure that can be supported by the targeted environment.

**Fig. 6.**
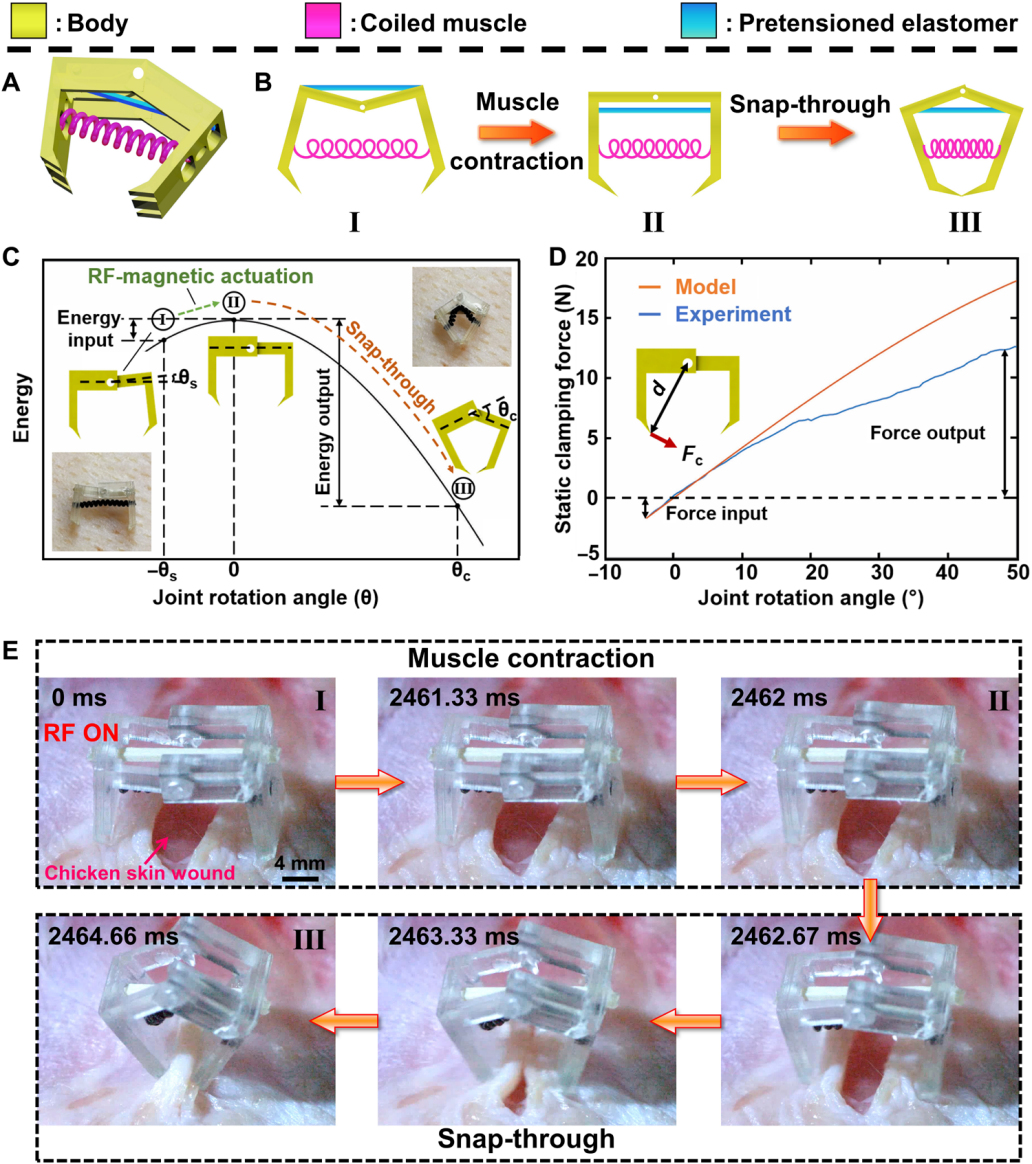
A wireless bistable clamper demonstration. (**A**) Schematic of the clamper designed with a bistable structure. (**B**) The sketch shows the clamping process under RF heating. (**C**) The energy distribution in the clamping process with different joint angles. (**D**) The experimental and the simulated clamping force of the clamper with different designed joint angles. (**E**) The images show that the clamper device clamps an ex vivo chicken tissue wound. These image snapshots are obtained from movie S10.

In the fourth case, we demonstrated a wireless bistable clamper device (Fig. 6). The clamper is useful for wound clamping and tissue pinching in surgical operations. It was constructed by combining a bistable linkage structure with the proposed magnetically heated coiled muscle. The bistable structure acted as a force amplifier, which could further boost the output force, response speed, and actuation strain performance of the proposed coiled muscle. The design is shown in Fig. 6A, and the actuation mechanism is shown in Fig. 6B and movie S9. The fabrication details are shown in Materials and Methods. A theoretical model was built to guide the clamper design (see the details in the “Modeling of the bistable clamper” section). The mechanical energy of the clamper was stored in a prestretched elastomer embedded within the rigid linkage (represented by blue in Fig. 6, A and B), which could be tuned from low to high energy storage capacity through simple elastomer pretension or setting the stop angle in the linkage. This prestretched elastomer enabled two stable states (states I and III in Fig. 6C) for the clamper. When RF-magnetic heating was turned on, the clamper deformed toward the unstable state II, where the elastomer stored the most energy. Once it bypassed the unstable state, it rapidly snapped to the other stable state III. The coiled muscle selected for this design had a force exertion of 1 N (see Materials and Methods). As shown in Fig. 6D, the output force of the coiled muscle could be amplified to 14 N through the proposed bistable design. A more detailed explanation of the bistable design can be found in the “Modeling of the bistable clamper” section. By using such a design enhanced by the bistable structure, we demonstrated an ex vivo clamping of a wound on a chicken skin in Fig. 6E and movie S10. The process could be divided into two parts: muscle contraction and elastomer snap-through. When RF-magnetic heating was turned on, muscle contraction started, driving the clamper from the stable state I to unstable state II, followed by a rapid snap to the stable state III within 2.7 ms. Furthermore, the bistable structure conferred the design with two additional capabilities. First, the snap-through of the bistability enabled much faster actuation than the coiled muscle alone. Second, the structure was also used to amplify the actuation strain of the muscle. Therefore, such a combination enabled a wider design space for future miniature medical device applications. In the current design, the clamping device is one-time actuation owing to the presence of the bistable structure. However, the target function of this clamper device is wound clamping and tissue pinching. Therefore, in most cases, this clamper does not need to be reopened. Even for the commercial tethered clamper (such as SureClip Hemoclips), it also exhibits one-time actuation. To achieve the reversible function, we can introduce an additional actuator, such as a shape memory material, to switch it back to the open state, as shown in fig. S12.

**Fig. 7.**
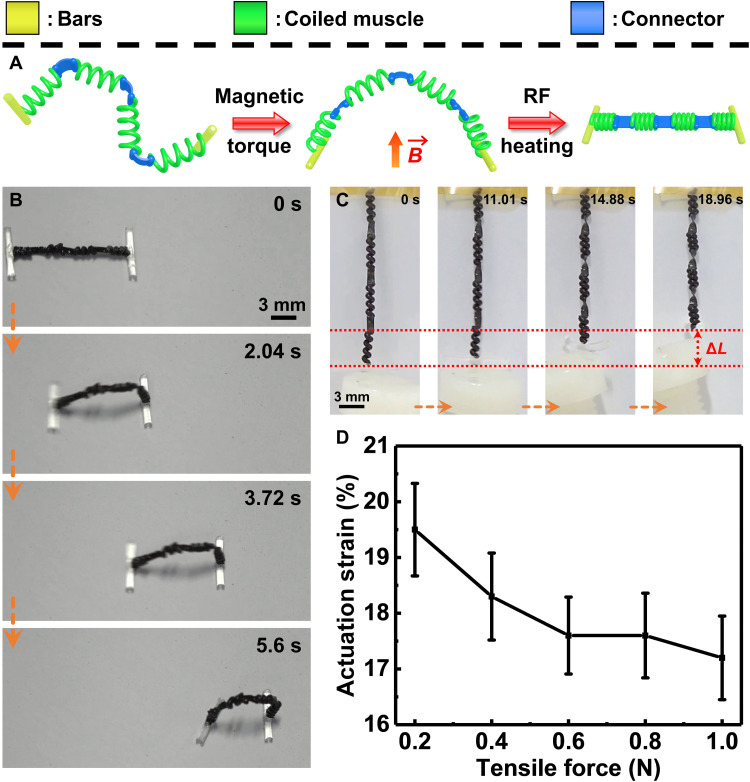
A multilinked coiled muscle. (**A**) Schematic shows the bending of the multilinked coiled muscle under magnetic torque and the contraction behavior under RF magnetic heating. The multilinked coiled muscle was encoded with a magnetization profile. This multilinked coiled muscle was composed of two bars and four coiled muscles linked by the connector. (**B**) The images show the walking behavior of the multilinked coiled muscle under magnetic torque and gradient. (**C**) Video snapshots indicate the contraction performance of the same multilinked coiled muscle under RF magnetic heating. (**D**) Actuation strain of this multilinked coiled muscle under different tensile forces. Images in (B) and (C) are obtained from movie S11.

In the last demonstration, we showed a multilinked coiled muscle with both programmable magnetic deformations as our previous research (*1*, *4*, *23*, *24*) and high force output (Fig. 7). To encode the magnetization profile on the coiled muscle, first, an additional sheath was coated on the outer surface of the precursor fiber. This magnetization sheath is composed of PDMS and NdFeB microparticles. After the modification (fig. S13), the artificial muscle has a total of three sheaths. It has to be pointed out that the protective sheath is essential as it binds with the magnetization sheath better than the active sheath (Crystal Clear). After the artificial muscles were made, four pieces (~4 mm) were linked in series by the connector, as shown in Fig. 7A. The connector is composed of PDMS and NdFeB microparticles. The connector is softer than the artificial muscle and therefore can amplify the magnetic deformation. Last, two bars are added to both sides of the multilinked coiled muscle to prevent the rotation of the coiled muscle under RF-magnetic heating. As shown in Fig. 7A and fig. S14, after magnetization, the multilinked coiled muscle can realize deformation under external magnetic torque. By using both magnetic torque and magnetic gradient, this multilinked coiled muscle exhibits a “walking” behavior, shown in Fig. 7B and movie S11. The moving direction of this multilinked coiled device can be controlled by the external magnetic field. It can move both forward and backward (fig. S14 and movie S12). In addition, when both sides are constrained from rotation but allowed to move axially (Fig. 7C and movie S11), this multilinked coiled muscle also exhibits contraction behavior with 19.5% actuation strain (Δ*L*) under 0.2 N load force. The maximum tensile force of this coiled muscle is about 1 N with 17.3% actuation strain. The maximal force is limited by the NdFeB-PDMS connector as it breaks when the tensile force exceeds 1 N. With these capabilities, the coiled muscle can potentially realize more diverse manipulation behaviors and medical functions in addition to its high force output and work capacity (*1*, *22*–*24*, *47*, *48*).

## DISCUSSION

The current coiled muscle can be improved in several directions. First, the current muscle core is a commercial nylon wire. It limits the potential to make miniature actuators. In nature, *Vorticella* can exhibit similar contraction behavior with a biological fiber of 2.6 μm in diameter (*49*). To address this, electrospinning can be used to reduce the diameter of the nylon core for a miniature actuator (*50*).

Second, the surface temperature of the coiled muscle can be further tuned for different applications. However, effects of the elevated temperature depend on the specific application. For the current coiled muscle, we have designed a protective insulation sheath on the outermost layer of the precursor fiber by using the PDMS. As shown in fig. S3, the surface temperature was reduced as increasing the thickness of the protective sheath. The surface temperature of the current coiled muscle was reduced to below 60°C. In addition, the surrounding temperature quickly drops with the distance away from the coiled muscle. As shown in fig. S3C, the temperature drops to 30°C even with a 2-mm separation from the coiled muscle. In general, normal cells can withstand temperatures of up to 42° to 45°C (*51*). For the scissor, driller, and clamper devices, owing to the presence of the printed frame, the coiled muscle is not in direct contact with the tissue. As shown in Figs. 4, 5, and 6, the distances between the coiled muscle and the tissue are approximately 5 mm, 4 mm, and 1 cm for the scissor, driller, and clamper device, respectively. Thus, the temperature on the tissue is lower than 30°C, causing no damage. For the suturing device and the multilinked device, the targeting application is to close the wound, which is in direct contact with the tissue. In this scenario, the high temperatures may benefit the wound closure during the suturing process through hyperthermia (*52*). Such a topic is worthy of further investigation in the future. Last, if there is a demanding requirement to further reduce the surface temperature of the coiled muscle, it can be achieved by increasing the thermal insulation efficiency of the protective sheath. The thermal conductivity of PDMS elastomer is 0.16 W/mK (*53*), which can be switched by cellulose (thermal conductivity is 0.04 W/mK) or polyurethane (thermal conductivity is 0.032 W/mK) (*54*).

Third, although the fast response is not essential in the proof-of-concept application demonstrations, such as suturing, scissor, driller, clamper, and multilinked actuator, it could be desirable for some future applications. For the current coiled muscle, the response speed can be increased by choosing materials with a lower glass-transition temperature, such as polycaprolactone (*55*). For these materials, external insulation sheath (protective sheath) is no longer required, consequently increasing the efficiency of heat dissipation.

## MATERIALS AND METHODS

### Precursor fiber fabrication

The precursor fiber was fabricated by the droplet-coating technique. In detail, 640 mg of Fe_3_O_4_ nanoparticles (50 to 100 nm particle size, Sigma-Aldrich company) and 63 mg of GO (Sigma-Aldrich company) were first added into 1.5 g of mixed Crystal Clear 202 resin [Smooth-On; the initial modulus (at ε = 1%) after annealing was measured to be 1.04 GPa, and the volume expansion ratio was 29.5% at 120°C]. Its base and curing agent (w/w 10:9) are stirred for 1 min. Next, the above mixture was degassed for 5 min. Then, a Nylon 6,6 fiber (Goodfellow) was immersed vertically into the above mixture and then drawn out of the uncured elastomer pool and cured by rapid heating at ~100°C. This step could be repeated multiple times to achieve the desired thickness (fig. S15). After drying at 30°C for 6 hours, the above fiber was then immersed vertically into a freshly mixed PDMS base and curing agent (w/w 10:1, Sylgard 184, Dow Corning). The fiber was then drawn out from the uncured PDMS pool and cured by heating at ~90°C. Last, after drying the above fiber at 30°C for 8 hours, the precursor fiber was obtained.

### Coiled muscle fabrication and characterization

The coiled muscle was obtained by the continuous twisting and coiling process. In a typical experiment, a 200-g weight was hung under one end of the precursor fiber, and the other was fixed to the shaft of a rotating motor. This configuration only allowed the fiber to rotate. After the fiber got sufficient turns and could no longer take more twists, the fiber started to coil on its axis. Next, the coiled muscle was stretched with a 32% prestrain. Then, the fully coiled fiber was annealed above ~180°C for 4 hours and evenly cooled down to room temperature to obtain the resulting coiled shape. Last, the coiled muscle was trained for actuation at 120°C with a 200-g load until consistent actuation is obtained. The SEM measurement was performed on an LEO-1530-VP SEM. The RF-magnetic heating system used in the current study was EASY HEAT 8310 (Ambrell Induction Heating Solutions). The charge-coupled device images and videos were captured by using a SONY DSC-RX10III digital camera. The uniform magnetic field was provided by a vibrating sample magnetometer (VSM, EZ7, Microsense).

### Thermal characterization of the protective layer

We recorded the temperature change of the coiled muscle with and without the outermost protective PDMS sheath under RF-magnetic heating by using a FLIR-A300 camera (FLIR Systems Inc.). As can be seen from fig. S3, the temperature of the coiled muscle without the PDMS sheath rises from 22.7° to 121°C in the air before and after RF-magnetic heating. This is because of the magnetically induced thermal effect of Fe_3_O_4_ nanoparticles (*27*). When the coiled muscle was coated with a PDMS sheath (fig. S3), the surface temperature decreased with the increasing thicknesses of the PDMS sheath due to the thermal insulation effect of the PDMS (*56*). While there was fracture during the twisting and coiling process, if the PDMS sheath thickness was higher than 200 μm, the minimum surface temperature of the coiled muscle was 60°C.

### Measurement of the muscle actuation force, torque, and actuation strain

The contractile actuation force of the coiled muscle shown in fig. S5 was measured directly by the Instron tensile measurement machine (INSTRON 5942). As shown in fig. S16A, the coiled muscle was prestretched by ~0.1%, and its two ends were directly clamped on the Instron machine’s bottom and top gripper. These two grippers were fixed without any further displacement. The tensile actuation force caused by the muscle contraction could thus be obtained. The torsional torque shown in Fig. 2 (C and F) was tested with a similar process. As shown in fig. S16B, one side of the muscle was fixed on the substrate. The other side was connected with the printed bar and tethered on the Instron’s gripper. Since it is difficult to use the Instron machine and RF-magnetic heating system at the same time, the heat gun (HE 20-600, Metabo) was replaced to heat the coiled muscle, and a thermal camera was used to monitor the temperature. We have compared the actuation strain of the coiled muscle under various loads when heated by heat gun and RF-magnetic heating. As shown in fig. S17, there is a negligible difference in the actuation strain between these two methods. It indicates that a heat gun is also useful for measuring the performance of this coiled muscle. The results of actuation strain shown in Fig. 2 (A and D) were characterized under RF-magnetic heating by applying nonrotating loads on the bottom end (fig. S16C), which provided the tensile force here. Each point in Fig. 2 (A and D) represents the maximum actuation strain of the coiled muscle during the contraction process under the corresponding loading force.

### Calculation of the work capacity

The work capacity shown in Fig. 2 (B and E) was defined as the product of actuation strain and tensile force from Fig. 2 (A and D, respectively), i.e.

$$\text{Work capacity}=\frac{l\times \text{actuation strain}\times \text{tensile force}}{m}$$

where *l* and *m* are the length and mass of the coiled muscle, respectively. The actuation strain is negatively correlated to the actuation stress. Consequently, the work capacity exhibits peaks (called the “load-optimized work capacity”) in Fig. 2 (B and E).

### Design of the bistable clamper

The untethered bistable clamper presented here was constructed by combining a bistable linkage structure with the proposed magnetically heated coiled muscle (Fig. 6A). It consisted of a bistable linkage structure with an embedded prestretched elastomer and the coiled muscle. The 3D-printed bistable clamper was composed of two rigid hinged linkages. The clamping force of the clamper without connecting the prestretched elastomer was measured to be ~1 N. The prestretched elastomer that connects two ends of the bistable mechanism enabled the storage and release of the potential strain energy.

### Calculation of the force and work capacity of the magnetic soft actuator

In this section, we build a simplified model to study how much work capacity a typical magnetic soft actuator could generate. As shown in fig. S1, we assume that the actuator has the residual magnetic flux density aligned with its longitudinal direction, one end of the beam is fixed while the other end is under a constant external load, and the beam is applied with a uniform magnetic field along the vertical direction. All these assumptions tend to generate a relatively large magnetic torque and thus result in a relatively large end-effector reaction force. This can help us predict, from the power perspective, the maximum capacity of a typical magnetic soft actuator in mechanical output.

When the magnetic field is not applied, the total potential energy of the actuator with the free end loaded (the convex shape shown in fig. S1) is

$$U_{t}=\int_{V}\frac{1}{2}\mathrm{EI}{Κ}^{2}\mathrm{dV}+\mathrm{Fd}$$ (1)

where the first term represents the bending energy in the soft actuator, and the second term represents the work done by the external force, *F*. *EI* is the bending stiffness of the soft bending actuator with *E* being Young’s modulus of magnetic soft composite and $I=\frac{1}{12}{\mathrm{wt}}^{3}$ being the second moment of inertia. $K\approx \frac{\theta }{L}$ is the approximate curvature in the soft actuator (where θ is the bending angle of the actuator). $d=L\frac{1-\text{cos}(\theta )}{\text{cos}(\theta )}$ is the deflection of the beam at the free end. It needs to be noted that, to simplify the model, we ignore the self-weight of the actuator and assume that the linear elasticity for the soft composite. We also assume that the beam is under pure bending and has a uniform curvature, which means θ is a constant. The equilibrium bending angle under the constant load of the beam, θ_0_, can be numerically solved by minimizing the total potential energy, i.e.

$$\frac{{\mathrm{dU}}_{t}}{d\theta }=0$$ (2)

With the magnetic field applied, the magnetic moment of the actuator (the concave shape shown in fig. S1) is

$$m=∭\frac{1}{\mu_{0}}B_{r}{\mathrm{dV}}_{m}$$ (3)

where *B_r_* is the residual flux density, μ_0_ is the permeability of the vacuum, and *V_m_* is the volume of the magnetic (NdFeB) microparticles. The potential energy induced by the magnetic actuation is defined as

$$U_{m}=-\vec{m}\cdot \vec{B}$$ (4)

where *B* is the external magnetic field. By substituting Eq. 3 into Eq. 4, we can get

$$U_{m}=-B\frac{B_{r}}{\mu_{0}}\cdot \mathrm{wt}\int_{L}\text{cos}(90{}^{\circ}-\varphi )\mathrm{dl}$$ (5)

where *w*, *t*, and *L* are the width, thickness, and length, respectively. φ is the angle between the magnetic moment and the horizontal direction. The work done by magnetic field in rotating the actuator from the loaded state (bending angle −θ_0_) to the actuated state (bending angle θ) is

$$U_{m}=B\frac{B_{r}}{\mu_{0}}\cdot \mathrm{wt}\int_{0}^{\theta_{0}}\text{sin}(\varphi )\frac{L}{\theta_{0}}d\varphi -B\frac{B_{r}}{\mu_{0}}\cdot \mathrm{wt}\int_{0}^{\theta }\text{sin}(\varphi )\frac{L}{\theta }d\varphi$$ (6)

Then, the total potential energy of the actuator is

$$\begin{array}{c} U_{t}=B\frac{B_{r}}{\mu_{0}}\cdot \mathrm{wt}\int_{0}^{\theta_{0}}\text{sin}(\varphi )\frac{L}{\theta }d\varphi -B\frac{B_{r}}{\mu_{0}}\cdot \mathrm{wt}\int_{0}^{\theta }\text{sin}(\varphi )\frac{L}{\theta_{0}}d\varphi +\\ \int_{V}\frac{1}{2}\mathrm{EI}(K^{2}-K_{0}^{2})\mathrm{dV}+F[d-(-d_{0})] \end{array}$$ (7)

where *K*_0_ and *d*_0_ are the curvature and deflection of the soft actuator at loaded state, respectively. *K* and *d* are the curvature and deflection of the soft actuator at actuated state, respectively. The equilibrium bending angle upon actuation of the beam can be numerically solved by $\frac{{\mathrm{dU}}_{t}}{d\theta }=0$. With the obtained θ_eq_, we can calculate the work capacity of the actuator by

$$w=\frac{F[d-(-d_{0})]}{L\cdot w\cdot t}$$ (8)

With this equation, we can predict the work capacity of the actuator as a function of the magnetic field as shown in fig. S2.

In fig. S2, we use *F* = 60 μN, *E* = 200 kPa, *L* = 3 mm, *w* = 1 mm, *t* = 0.1 mm, *B_r_ =* 0.84 T, and *V_m_/V* = 0.3 (NdFeB particles/Ecoflex in volume ratio). The result shows that the work capacity increases monotonically first with the magnetic field and then approaches a plateau at *B* = ~7.8 mT, exhibiting a highly nonlinear behavior. Further increasing the magnetic field beyond *B* = ~7.8 mT does not substantially change the work capacity, which remains as a constant at ~2 × 10^−4^ kJ/kg. This plateau stage is due to the full alignment of the magnetic moment with the magnetic field. It needs to be noted that we choose *E* = 200 kPa because Young’s modulus (*E*) of the magnetic soft actuator is within the range of 6.6 to 200 kPa (*1*, *22*–*24*). Furthermore, we use *F* = 60 μN because an external load larger than 60 μN will lead to a huge deflection, or even collapse, of the soft beam at the loaded state (according to the numerical result by Eq. 2), thus preventing the actuator from functioning as designed and being useful. Note that the output force is obtained from solving the relation between the output force and the deflection of the magnetic soft actuator (Eq. 2) by using a numerical method (the corresponding Matlab code is shown in fig. S18).

It is observed that although the assumptions we made for the model (a large *B_r_*, a homogeneous magnetic moment, a perpendicular magnetic field to the residual magnetic flux density, and a saturated NdFeB-ecoflex volume ratio) tend to generate a large torque (i.e., high force) in the magnetic soft actuator, its maximum work capacity is still only ~2 × 10^−4^ kJ/kg, which is ~10^2^ weaker than skeletal muscle, and ~10^4^ weaker than the proposed magnetic artificial muscle. Unfortunately, in reality, a magnetic soft actuator normally has a heterogeneous magnetic moment, a smaller angle between the magnetic moment and magnetic field, less volume ratio of magnetic particles, and different boundary conditions (e.g., two ends are free), all of which tend to generate a smaller torque. Therefore, we expect their “real-life” work capacity to be much smaller than the result shown in fig. S2. This explains why we show a smaller work capacity for the magnetic soft actuator in Table 1.

### Calculation of tensile actuation strain, work capacity, and torsional torque

In this section, we have built a model to calculate the tensile actuation strain, work capacity, and torsional torque of the coiled muscle. For this trimorph muscle, Young’s modulus of the coiled muscle core (*E*_core_), active sheath (*E*_active_), and protective sheath (*E*_protective_) are measured after the annealing process, which are about 1.2 GPa, 1.04 GPa, and 1.5 MPa, respectively, i.e.

$$\begin{array}{c} E_{\text{core}}\approx E_{\text{active}}\\ E_{\text{core}}\gg E_{\text{protective}}\\ E_{\text{active}}\gg E_{\text{protective}} \end{array}$$ (9)

Therefore, to simplify the model, we assume this three-layer coiled muscle as one-layer helical spring, and we choose *E*_core_ = 1.2 GPa as the Young’s modulus. First, the dependence of the actuation strain (ε) and work capacity (*W*) on loading force (*F*) can be calculated from fig. S19. There are four states (I to IV) of the coiled muscle. States I and II represent the nonactuated states, and states III and IV are the actuated states. While States I and III are free states, that means no load is hung on the bottom end. Correspondingly, States III and IV are the loaded states. From the above figure, the actuation strain and the work capacity with loading force can be obtained

$$\varepsilon =\frac{∆y}{l}=\frac{y_{0}+y_{1}-y_{2}}{l}$$ (10)

$$W=\frac{F∆y}{m}=\frac{F(y_{0}+y_{1}-y_{2})}{m}$$ (11)

where *m*, *l*, *y*_0_, and Δ*y* are the mass, length, free actuation length, and load actuation length of the coiled muscle, respectively. According to Hooke’s law, $y_{1}=\frac{F}{k_{1}}$, $y_{1}=\frac{F}{k_{2}}$. Therefore, Eqs. 10 and 11 can be rewritten as

$$\varepsilon =\frac{y_{0}}{l}+\frac{F}{l}(\frac{1}{k_{1}}-\frac{1}{k_{2}})$$ (12)

$$W=\frac{Fy_{0}}{m}+\frac{F^{2}}{m}(\frac{1}{k_{1}}-\frac{1}{k_{2}})$$ (13)

where *k*_1_ and *k*_2_ represent the spring stiffness of the nonactuated and actuated coiled muscle, respectively. Therefore, the actuation strain and the work capacity are determined by the free actuation strain ($\frac{y_{0}}{l}$) and the change of inverse spring stiffness $\frac{1}{k_{1}}-\frac{1}{k_{2}}$. To finish the calculation, we have to derive the free actuation strain and the spring stiffness. For the free actuation strain ($\frac{y_{0}}{l}$), according to the coiled-driven actuation mechanism (*57*), it can be obtained from

$$\frac{y_{0}}{l}=\frac{l∆T}{N}$$ (14)

where *N* is the number of coils of the coiled muscle; Δ*T* is the torsional stroke, which can be calculated by the following equation

$$∆T=\frac{n}{l}-\frac{n_{0}}{l_{0}}$$ (15)

where *n* is the number of fiber turns for making the helix coiled muscle; *n*_0_ and *l*_0_ are the initial values. According to the previous literature (*58*), the changing fiber turns of the coiled muscle is caused by the fiber volume expansion in the actuation process. The relationship is

$$\frac{n}{n_{0}}={\left(\frac{V_{0}}{V}\right)}^{\frac{1}{2}}(\frac{h}{h_{0}}∙\frac{l^{2}-h^{2}}{l_{0}^{2}-h_{0}^{2}})$$ (16)

where *V* and *h* are the volume and length of the constructed fiber, respectively. Zero subscripts represent the corresponding initial values. We assume that the change of the fiber length in the tensile actuation process is negligible. Equation 16 can be simplified to

$$\frac{n}{n_{0}}\approx {\left(\frac{V_{0}}{V}\right)}^{\frac{1}{2}}=\frac{d_{0}}{d}$$ (17)

where *d* is the diameter of the constructed fiber. Therefore, by substituting Eqs. 15 and 17 to Eq. 14, the free actuation strain $\frac{y_{0}}{l}$ can be obtained as

$$\frac{y_{0}}{l}=\frac{n_{0}}{N}(\frac{d_{0}}{d}-1)$$ (18)

In addition, the spring stiffness of the coiled muscle is calculated by using Castigliano’s theorem (*59*)

$$k=\frac{d^{4}G}{8D^{3}N}$$ (19)

where *D* is the diameter of the coil structure, which we assume has a negligible change in the actuation process. *G* is the shear modulus, i.e.

$$G=\frac{E}{2(1+\delta )}$$ (20)

where δ is the Poisson’s ratio of the fiber. By combining Eqs. 12, 13, 18, 19, and 20, we can calculate the actuation strain and work capacity

$$\varepsilon =\frac{n_{0}}{N}(\frac{d_{0}}{d}-1)+\frac{16\mathrm{FN}}{\mathrm{El}}(1+\delta )(\frac{D^{3}}{d_{0}^{4}}-\frac{D^{3}}{d^{4}})$$ (21)

$$W=\frac{ln_{0}F}{\mathrm{mN}}(\frac{d_{0}}{d}-1)+\frac{16F^{2}N}{\mathrm{mE}}(1+\delta )(\frac{D^{3}}{d_{0}^{4}}-\frac{D^{3}}{d^{4}})$$ (22)

For the investigated coiled muscle, the corresponding parameters are listed below: the number of coils (*N*) is *N* = 80; the initial number of fiber turns (*n*_0_) is *n*_0_ = 278; the diameter of the constructed fiber (*d*) is *d* = 0.73 mm and the initial value (*d*_0_) is *d*_0_ = 0.65 mm; the length of the coiled muscle (*l*) is *l* = 11 cm; the diameter of the coil structure (*D*) is *D* = 1 mm. Here, to simplify the model, we choose Young’s modulus (*E*) and Poisson’s ratio (δ) from the nylon fiber core, i.e., *E* = 1.2 GPa, δ = 0.39, as parameters of the constituent fiber in the model. Therefore, we can predict the dependence of the actuation strain and the work capacity on loading force by using Eqs. 21 and 22 and the above parameters. As shown in fig. S20, we have compared the theoretical results and the experiment results to prove the correctness of our model.

The mismatch between the theoretical and experimental results can be explained as below. First, we assume that the trimorph structure to one-layer structure of the coiled structure and use the nylon fiber’s Young’s modulus and Poisson’s ratio as the parameter of the whole constituted fiber. Next, in this model, we did not consider the change of the fiber’s Young’s modulus between the actuated and nonactuated states. We just considered the diameter expansion of the constituted fiber and assumed that the fiber length and the diameter coiled structure have negligible change. Last, in the actuation process, intercoil contact is exhibited if the loading force is small, which is not the case in this model.

In addition, according to the previous literature (*58*), the torsional torque (τ) can be calculated by the following equation

$$\tau =∆n\frac{\mathrm{JG}}{l}$$ (23)

where *J* = πd^4^/32 is the polar second moment of area; ∆*n* = *n* − *n*_0_ is the changing number of fiber turns due to the fiber volume expansion in the actuation process. Combining Eqs. 17, 20, and 23, the torsional torque can be obtained as

$$\varepsilon =\frac{\pi d^{4}Gn_{0}}{32l}(\frac{d_{0}}{d}-1)=\frac{\pi d^{4}En_{0}}{64l(1+\delta )}(\frac{d_{0}}{d}-1)$$ (24)

This equation indicates that the torsional torque increases with increasing material’s Young’s modulus and the fiber diameter, following the same trend as in the experimental results in Fig. 2 (C and F).

### Modeling of the bistable clamper

As schematically illustrated in Fig. 6B, the working principle of the bistable clamper follows four steps. First, the elastomer is prestretched and attached to both ends of the linkages to enable bistability. The linkage-based structure with embedded prestretched elastomer results in an unstable state (state II in Fig. 6B), which has the maximum potential energy as shown in the schematic energy profile of Fig. 6C (state II) and a zero joint revolute angle θ. Second, after releasing the elastomer, the clamper rotates and deforms into a concave shape (Fig. 6B) and rests in one stable state (state I in Fig. 6C), which has local minimum energy at θ *= −*θ*_s_* (here, we set the angular position limit of the bistable mechanism to stop its rotational movement at preset stopping angles, θ*_s_*, as shown in Fig. 6C). Then, we attach a stress-free coiled muscle to connect the two arms of the clamper, as shown in Fig. 6B. Third, with the contraction of the coiled muscle upon magnetic heating, the clamper deforms toward the unstable state, where the elastomer stores the most energy. Fourth, when it bypasses the unstable state II, it rapidly snaps to the other stable state III with θ *=* θ*_c_* (θ*_c_* is the revolting angle at which the target tissue is clamped, Fig. 6C). By setting θ*_c_ >>* θ*_s_*, this stable state III has much lower potential energy than another stable state I.

Figure 6C schematically shows the energy landscape of the bistable actuator as a function of the bending angle θ. It shows one peak energy state (unstable state II) and two localized lower energy states (stable states I and III). The difference between the peak and the lower energy state defines the energy barrier. Here, we define the energy barrier from state I to state II as Δ*E*_1_ and the released energy from state II to state III as Δ*E*_2_. To enable the actuation from state I to state II, the muscle actuation should provide sufficient energy input to trigger the snap-through instability, i.e., *U*_in_ ≥ Δ*E*_1_. Once bypassing state II, the bistable system will rapidly snap to another stable state III, during which a huge amount of stored strain energy in elastomer will be quickly released, resulting in a large energy output Δ*E*_2_. It is observed that by setting θ*_c_* >> θ*_s_*, the energy output of this system will be much larger than input, i.e., Δ*E*_2_ >> *U*_in_ ≥ Δ*E*_1_, showing an energy/force amplifying the effect.

### Energy-based theoretical model and evaluation of the clamping force

We develop an energy-based theoretical model to understand the nonlinear behavior of the proposed bistable clamper, including the relationship among design parameters (e.g., linkage stop angle and prestretched strain in elastomer) and outputs (e.g., potential energy and output force), as shown below. The total potential energy of the bistable clamper is

$$U_{t}=U_{\text{muscle}}+U_{\text{elastomer}}$$ (25)

where *U*_muscle_ is the potential energy in the coiled artificial muscle and *U*_elastomer_ is the potential energy in the prestrained rubber. In our case, the rubber has a high modulus (~2 MPa) and is prestretched at a high strain; thus, its potential energy will be much larger than that in the twisting muscle, i.e., *U*_elastomer_ *>> U*_muscle_. In this case, the second term in Eq. 25 can be ignored. Then, we can rewrite Eq. 25 as

$$U_{t}=U_{\text{elastomer}}=\frac{1}{2}\int E\varepsilon^{2}\mathrm{dV}$$ (26)

where *E* is the Young’s modulus of the elastomer and *V* is the volume of the elastomer. ε is the strain of the elastomer at a given joint rotation angle θ as

$$\varepsilon =\text{cos}(\frac{\theta }{2})\cdot (1+\varepsilon_{\text{pre}})-1$$ (27)

where ε_pre_ is the prestretched strain of the elastomer at unstable state II. Then, we can rewrite Eq. 26 as

$$U_{t}=\frac{1}{2}\int E[\text{cos}(\frac{\theta }{2})\cdot (1+\varepsilon_{\text{pre}})-1){}^{2}]\mathrm{dV}$$ (28)

It should be noted that we assume the idealized linear elastic behavior in the homogenized continuous material despite the nonlinear deformation in the elastomer. On the basis of the total potential energy of the system, *U_t_*, in Eq. 28, the joint torque, *T*, of the bistable actuator can be obtained by

$$T=\frac{{\mathrm{dU}}_{t}}{d\theta }$$ (29)

The clamping force of the bistable actuator, *F_c_*, or the reaction force of the end-effector can be obtained by

$$F_{c}=\frac{T}{d}$$ (30)

where *d* is the distance between the clamping tip and the joint, as shown in Fig. 6D. On the basis of this equation, we plot the clamping force as a function of joint rotation angle, θ, as the orange curve shown in Fig. 6D. It shows that by setting θ*_s_ =* 4^o^, *E* = 2 MPa, and ε_pre_ = 400%, the force required to bypass the energy barrier is ~1.46 N. Once the onset of the snap-through instability occurs, the structure snaps and the corresponding output force can reach ~18.1 N at θ*_c_ =* 50^o^, showing a ~10.7 times amplification in output force. To validate the model, we experimentally examine the static output force of the bistable clamper as a function of bending angle through a quasi-static indenting test with the setup shown in fig. S21. Similar to the trend captured by the model, the experiment result shows that by increasing the difference between θ*_s_* and θ*_c_*, e.g., θ*_s_ =* 4^o^ and θ*_c_* = 50^o^, the system shows a huge force amplification by a factor of up to ~7.43 (the mismatch between the model and experiment is explained in detail below). To better understand the force amplification effect, we plot the corresponding output force/force input as a function of θ*_c_*/θ*_s_* in fig. S22. It shows that the force-improved ratio of the bistable system increases monotonically with θ*_c_*/θ*_s_*, indicating that we can simply boost the output force/input force from 1.0 to 7.4 by simply tuning θ*_c_*/θ*_s_* from 1 to 13. In addition to the joint angle, the energy and force landscape of the bistable system can also be tuned by prestretched strain, ε_pre_, in the elastomer, as shown in fig. S23, where the clamping force and energy output increased markedly with ε_pre_.

Our analytical model and the experiment results only capture the static (or quasi-static) response of the bistable systems. If we include the dynamic effects of the system during the snap-through process, its output force would be much larger than the force we modeled (and measured) because a fast snap-through instability normally contributes to a larger dynamic force. The mismatch between the model and the experiment results shown in Fig. 6D is explained as below. First, in the model, the potential energy of the twisting muscle is ignored. In reality, the strain energy in twisting muscles will increase at a large bending angle. This explains why only a small mismatch between model and experiment is observed at small θ, but a large discrepancy is observed at large θ. Next, in the model, we assume that the friction at the joint is zero. In reality, the joint friction is not zero, and it increases with ε_pre_. Third, we assume linear elasticity for the elastomer in the model. According to our measured stress-strain curve of the elastomer, as shown in fig. S24, it shows a highly nonlinearity despite a nearly constant slope at a small strain.
