# Supplementary material for: Miniature coiled artificial muscle for wireless soft medical devices
Source: Sci Adv. 2022 Mar 11;8(10):eabm5616. doi: 10.1126/sciadv.abm5616 (PMC8916729; doi:10.1126/sciadv.abm5616)
Supplement: Supplementary file 1 — Figs. S1 to S24 [file sciadv.abm5616_sm.pdf]

Science Advances
AAAS

Supplementary Materials for

Miniature coiled artificial muscle for wireless soft medical devices

Mingtong Li, Yichao Tang, Ren Hao Soon, Bin Dong, Wenqi Hu*, Metin Sitti*

*Corresponding author. Email: wenqi@is.mpg.de (W.H.); sitti@is.mpg.de (M.S.)

Published 11 March 2022, *Sci. Adv.* **8**, eabm5616 (2022)
DOI: 10.1126/sciadv.abm5616

The PDF file includes:

Figs. S1 to S24
Legends for movies S1 to S12

Other Supplementary Material for this manuscript includes the following:

Movies S1 to S12

Supplementary Figures

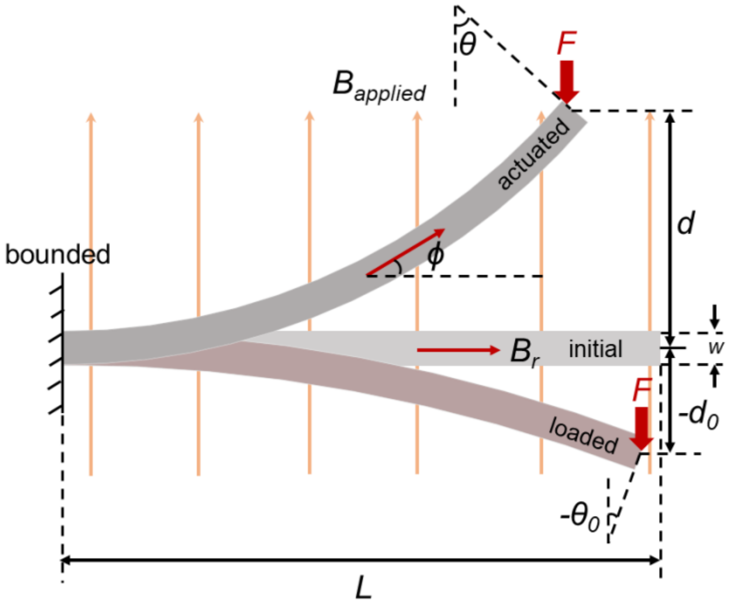

Fig. S1. Modeling of the magnetic soft actuator. Schematic of the actuation of a magnetic soft actuator.

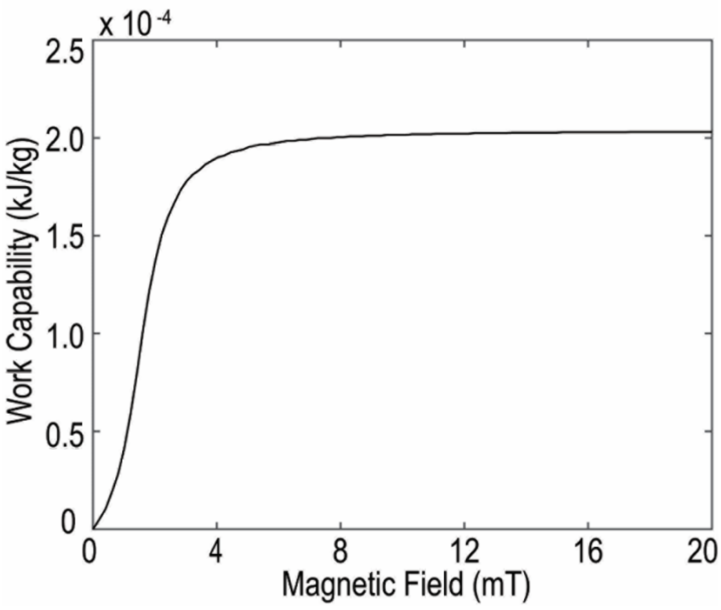

Fig. S2. Calculation of the magnetic soft actuator work capacity. The theoretical work capacity of the magnetic soft composite actuator as a function of the applied magnetic field.

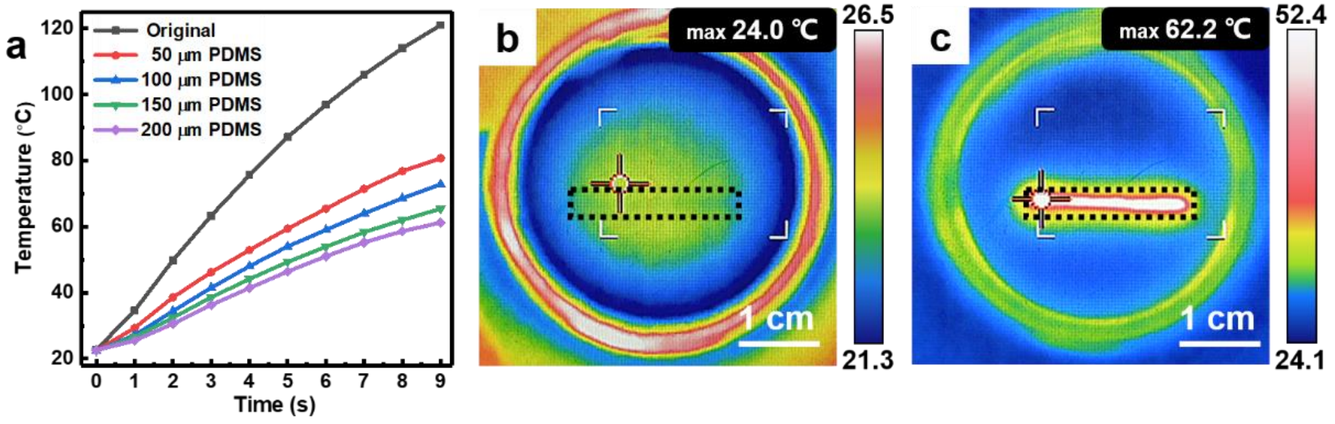

Fig. S3. Thermal characterization of the coiled muscle. (a) The surface temperature changes of the coiled muscle with different coated PDMS thickness under RF heating. Infrared thermal images of the coiled muscle with 200 μm PDMS sheath thickness (b) before and (c) after RF heating. Note: the black dashed rectangle indicates the position of the coiled muscle.

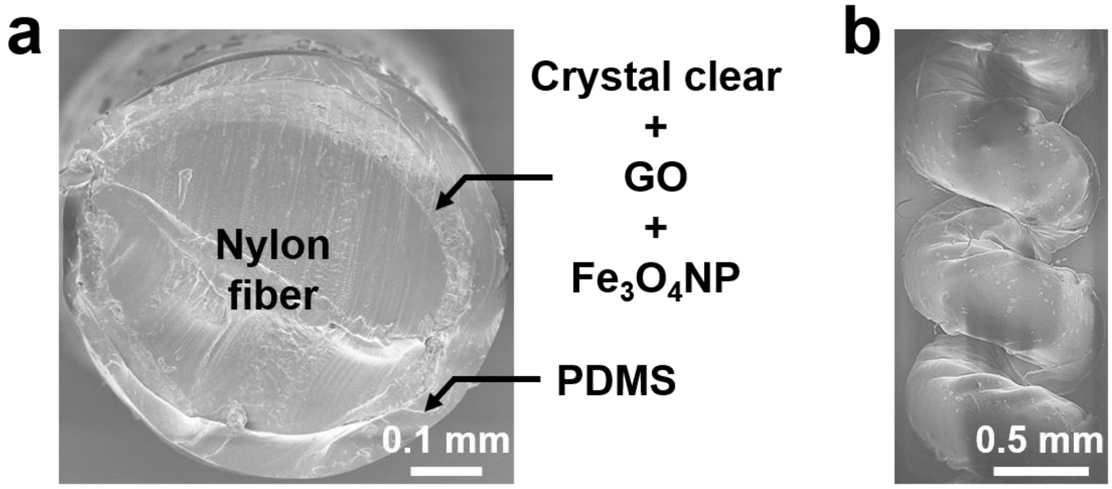

Fig. S4. SEM morphology characterization. (a) The cross-sectional structure of the precursor fiber and (b) the final coiled muscle.

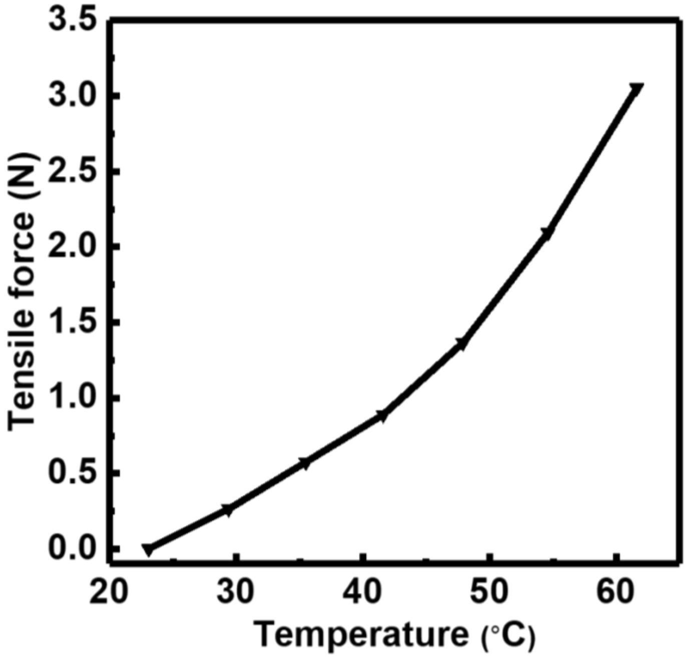

Fig. S5. Output force characterization. The tensile force of the coiled muscle with a constant strain with 0.1% under heating.

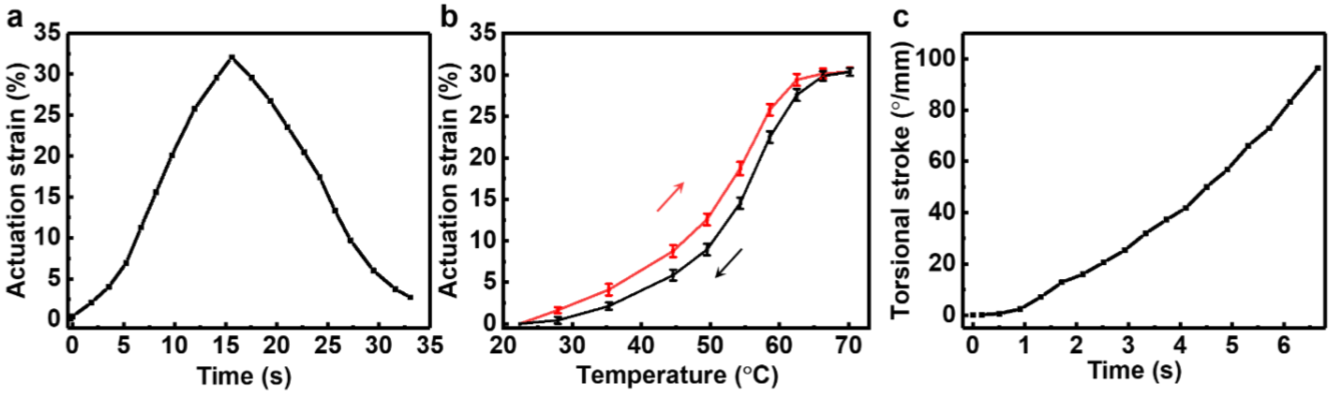

Fig. S6. Tensile strain and torsional stroke of the coiled muscle. The change of tensile actuation strain versus (a) time and (b) temperature in the contractile actuation shown in Movie S1. (c) The torsional stroke in the torsional actuation shown in Movie S2. Note: in (b), the red line and black line represent the temperature increasing and decreasing processes, respectively.

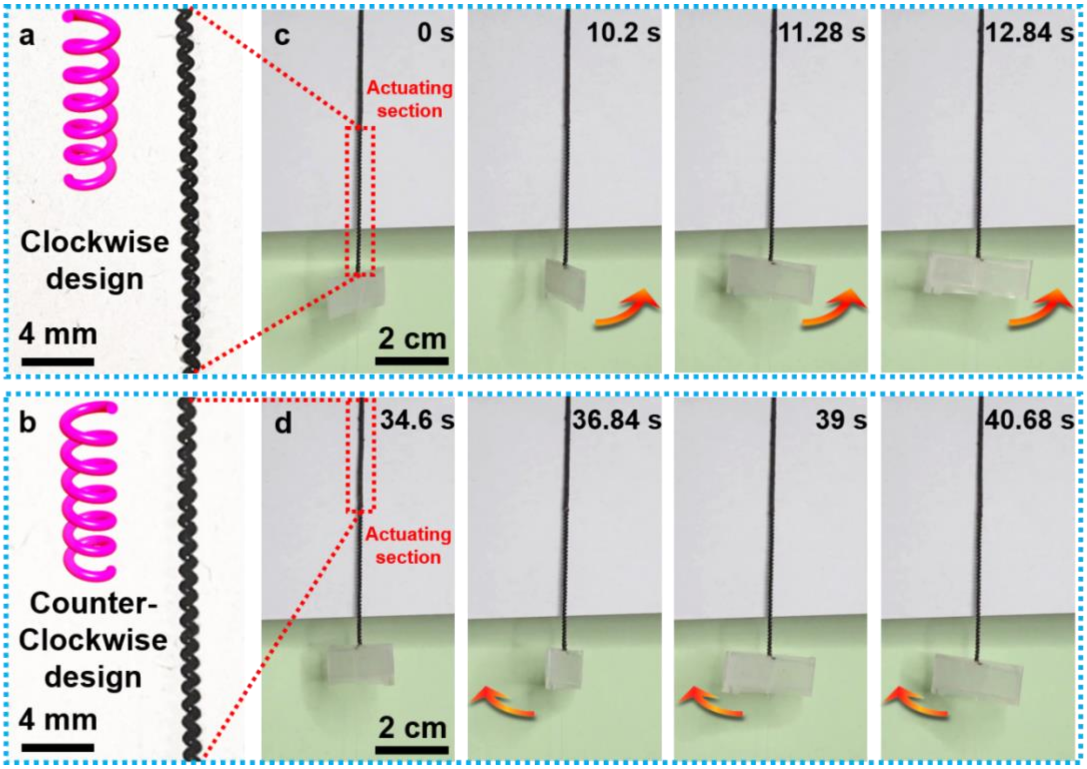

Fig. S7. Reversible torsional actuation. The coiled muscle with (a) lower clockwise section and (b) upper counter-clockwise section. The corresponding (c) counter-clockwise rotation and (d) clockwise rotation of this coiled muscle under RF heating. The images in (c) and (d) are obtained from Movie S3.

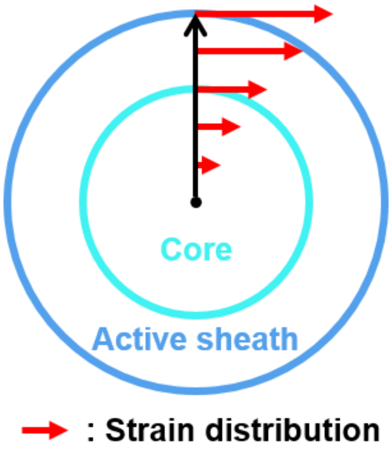

Fig. S8. The strain distribution of the coiled muscle (cross-sectional view).

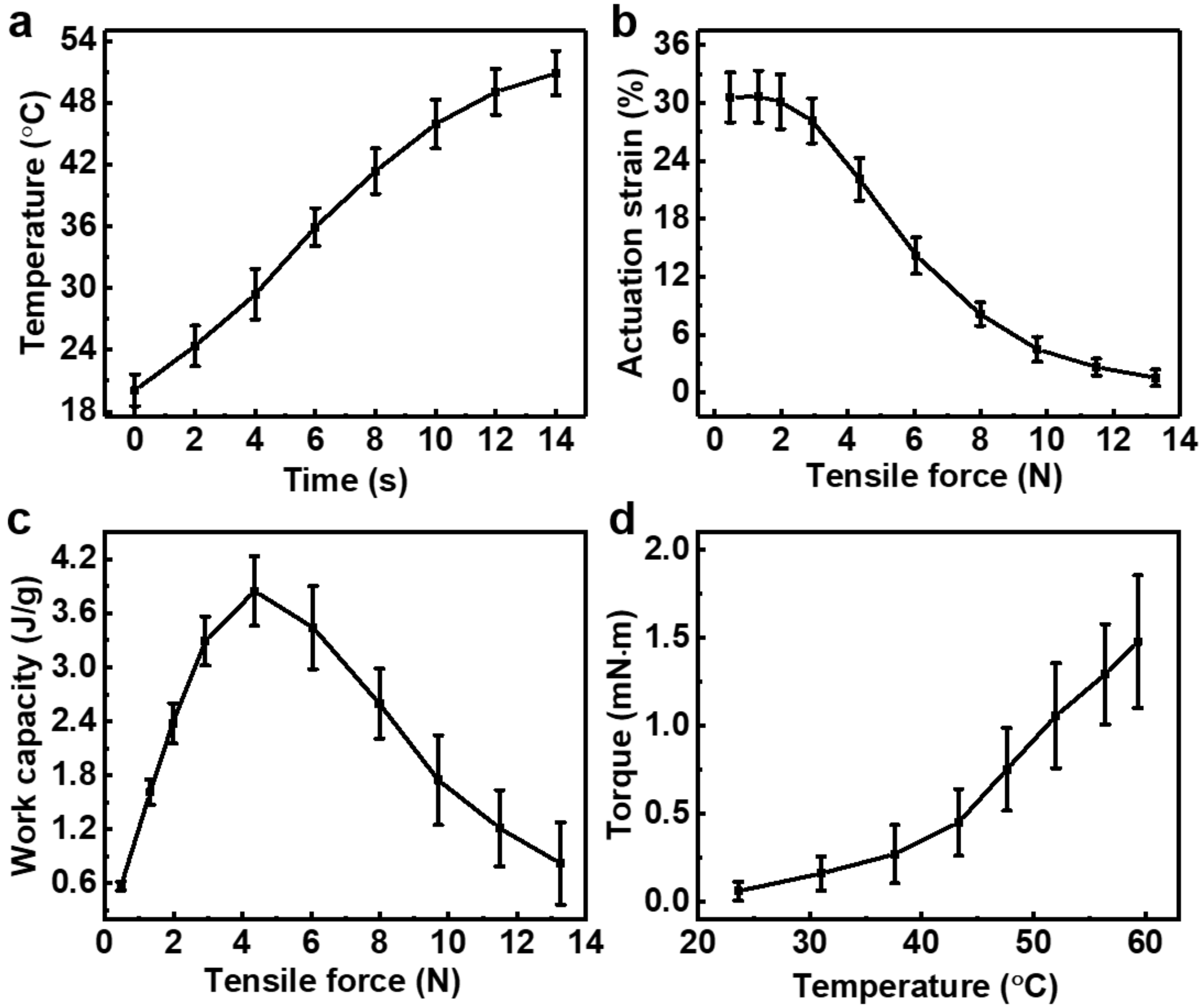

Fig. S9. Characterization of the coiled muscle performance in deionized water. (a) The temperature changes of the coiled muscle under RF heating. (b-c) The actuation strain and the calculated work capacity of the coiled muscle with different tensile forces. (d) The torsional torque of the coiled muscle under RF heating. Note, this coiled muscle is composed of 4 wt% GO and 200 μm active sheath.

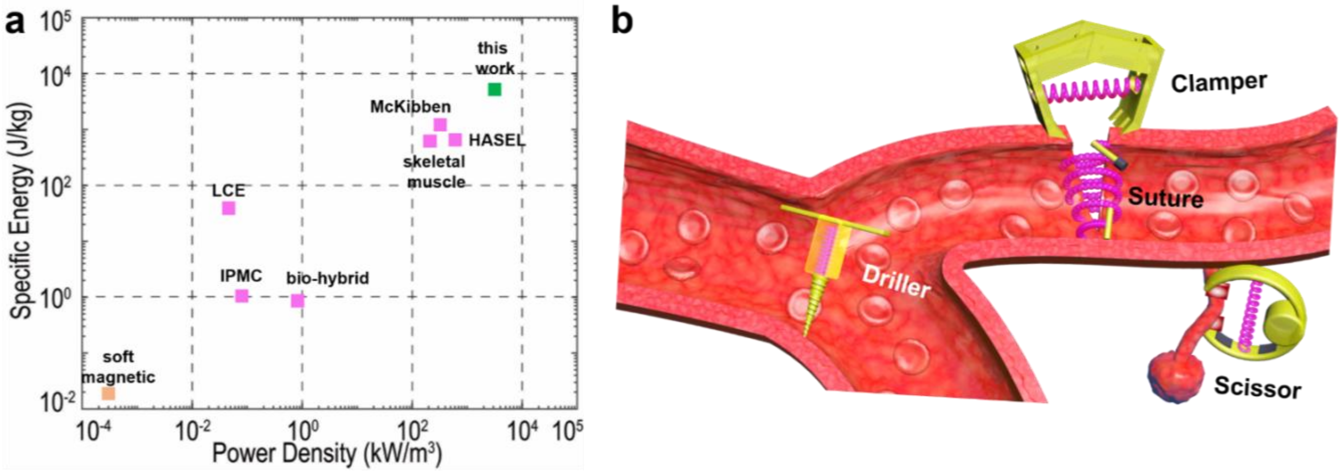

Fig. S10. Performance evaluation and application prospects of the coiled muscle. (a) Comparison of different types of soft actuators in terms of work capacity and power density. (b) The schematic shows the coiled muscle actuator engineered into a suturing device, scissor device, driller device, and a clamper device toward biomedical applications.

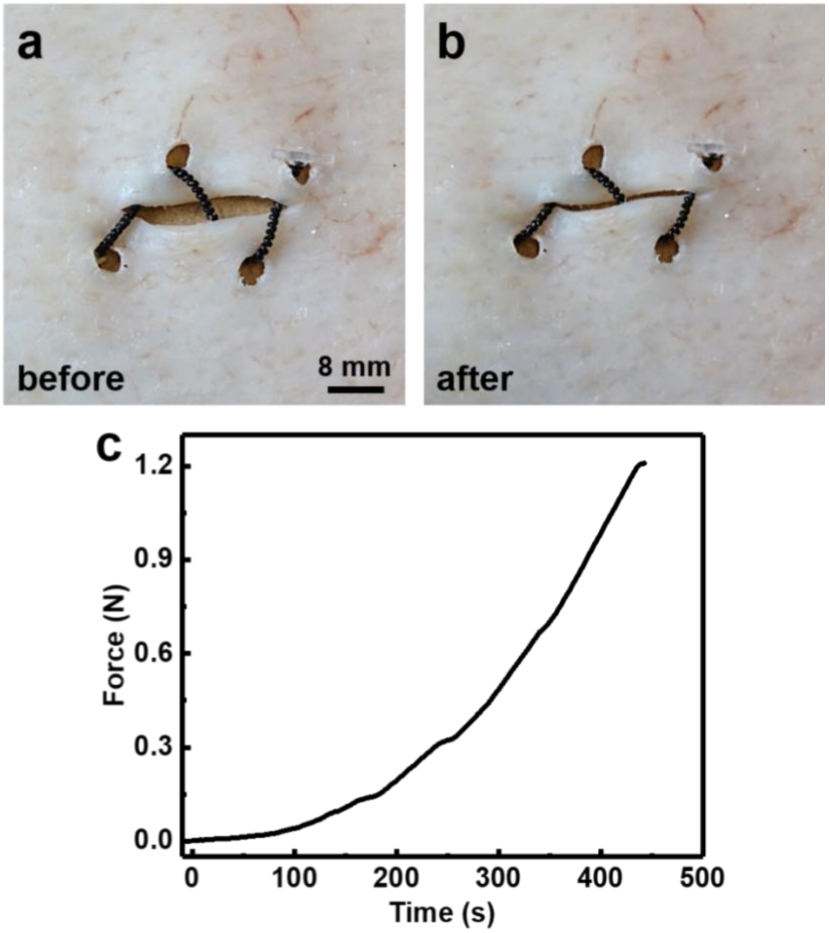

Fig. S11. Force tests for the suturing device. Photos of the pigskin wound (a) before and (b) after closing by Instron machine pulling. (c) The pulling force for this pulling process.

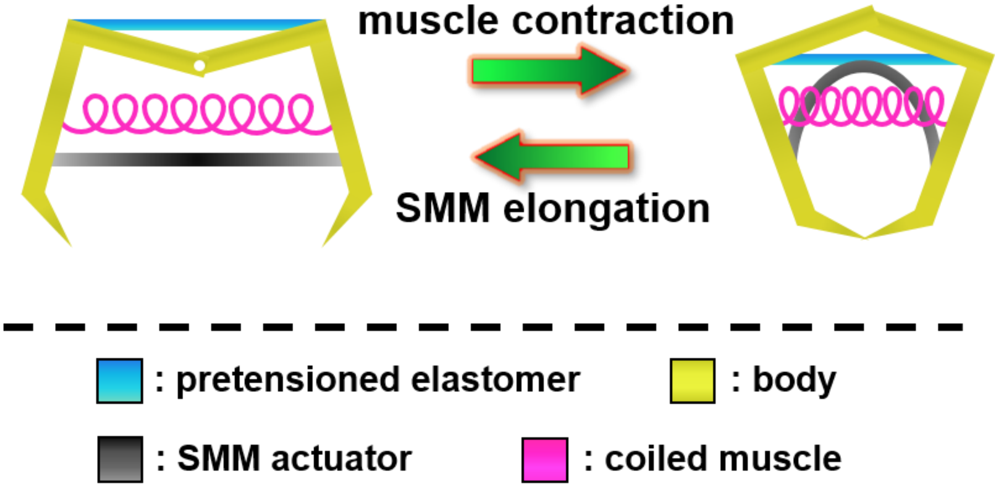

Fig. S12. Design of a reversible clamper device. Schematic showing the reversible clamping process cooperated with the muscle contraction and shape memory material (SMM) elongation.

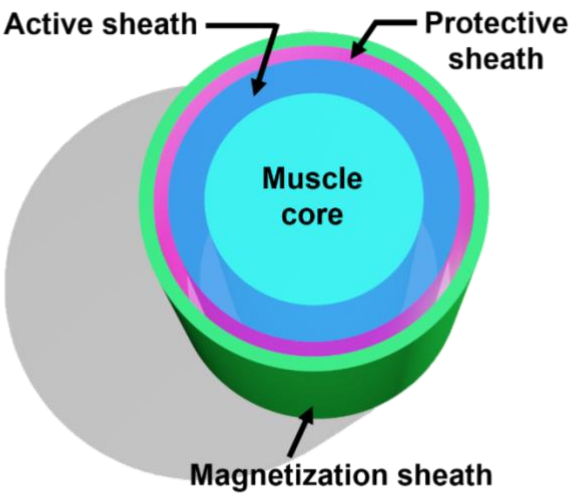

Fig. S13. Precursor fiber design of the multi-linked coiled muscle. The schematic shows the cross-sectional structure of the precursor fiber after coating the magnetization sheath.

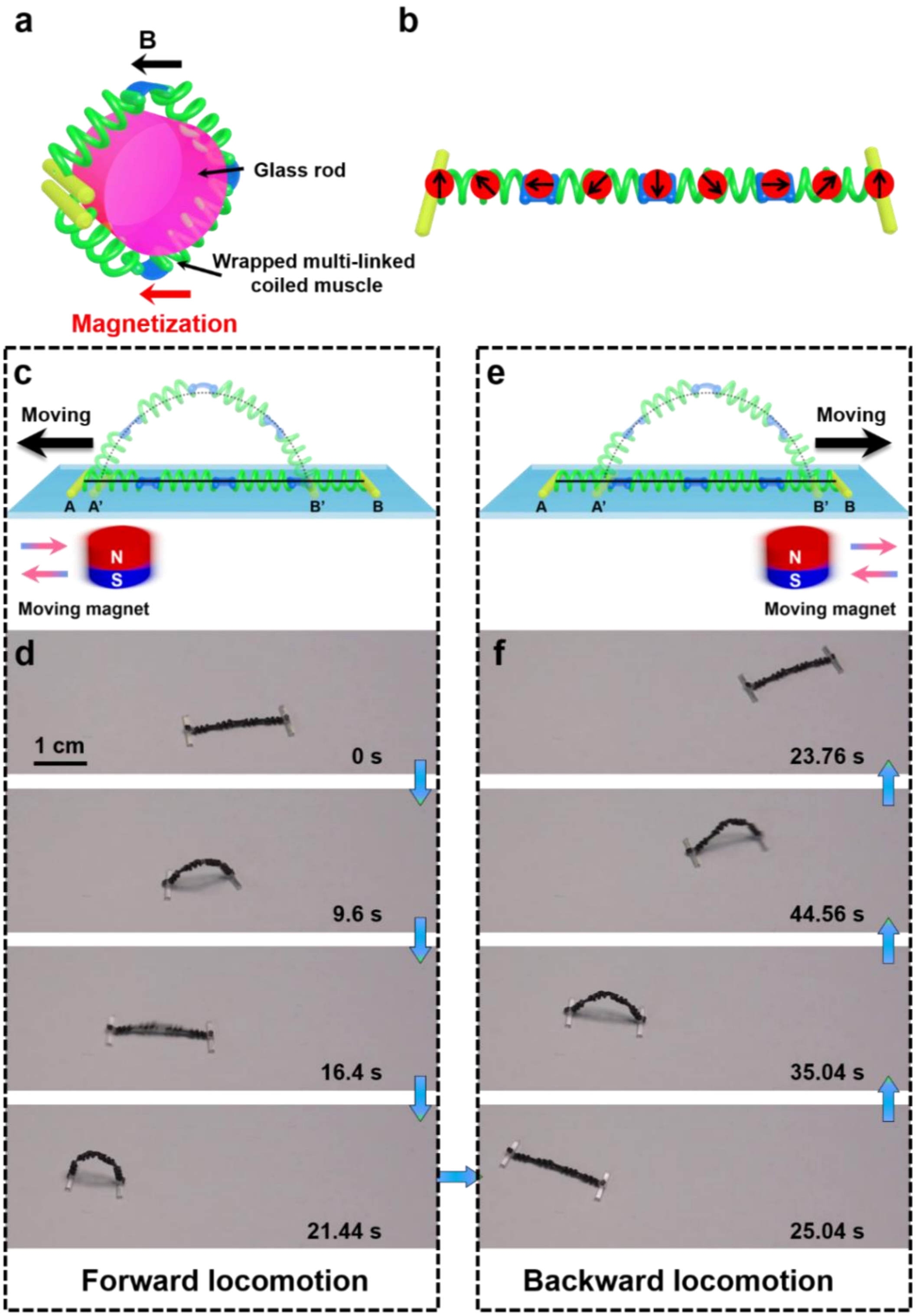

Fig. S14. Surface walking locomotion of the multi-linked coiled muscle. Schematic (a) showing a multi-linked coiled muscle wrapped around a cylindrical glass rod and magnetized by a uniform 1.8 T magnetic field and (b) the corresponding distribution of magnetization directions. The (c-d) forward and (e-f) backward locomotion of the multi-linked muscle under external magnetic field. The pictures of d and f are obtained from the screenshot of the Movie S12.

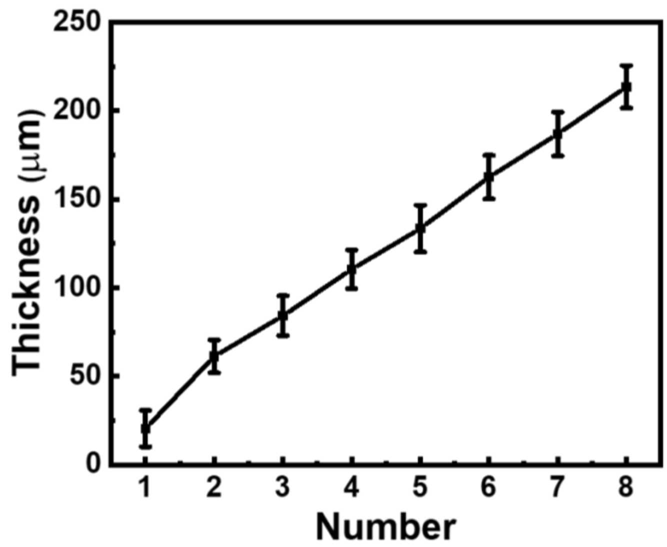

Fig. S15. Active sheath thickness characterization. The thickness of active sheath versus the times of droplet-coating technique.

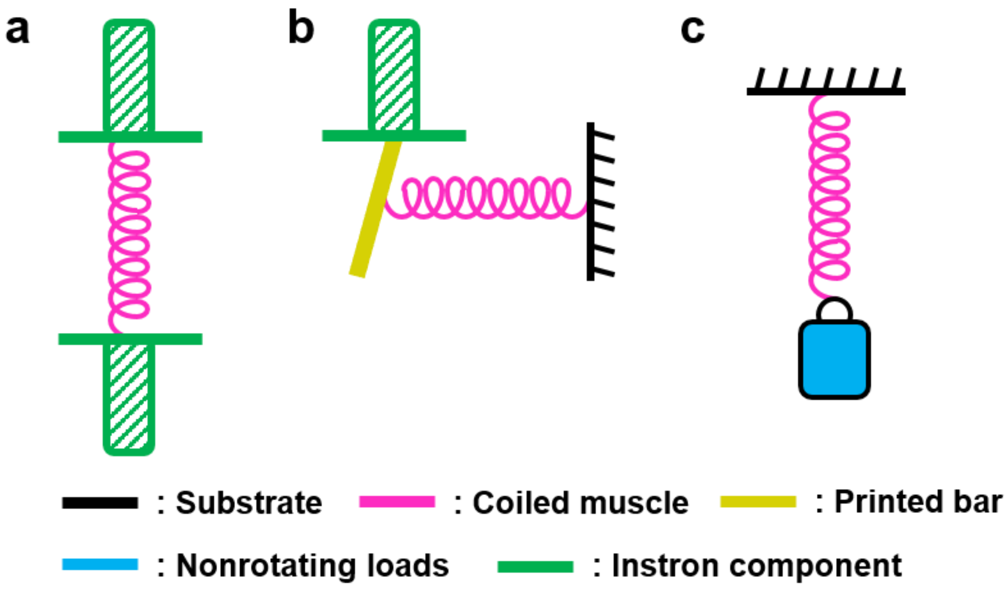

Fig. S16. Schematic of measurement. The measurement of (a) tensile actuation force, (b) torsional torque, and (c) actuation strain.

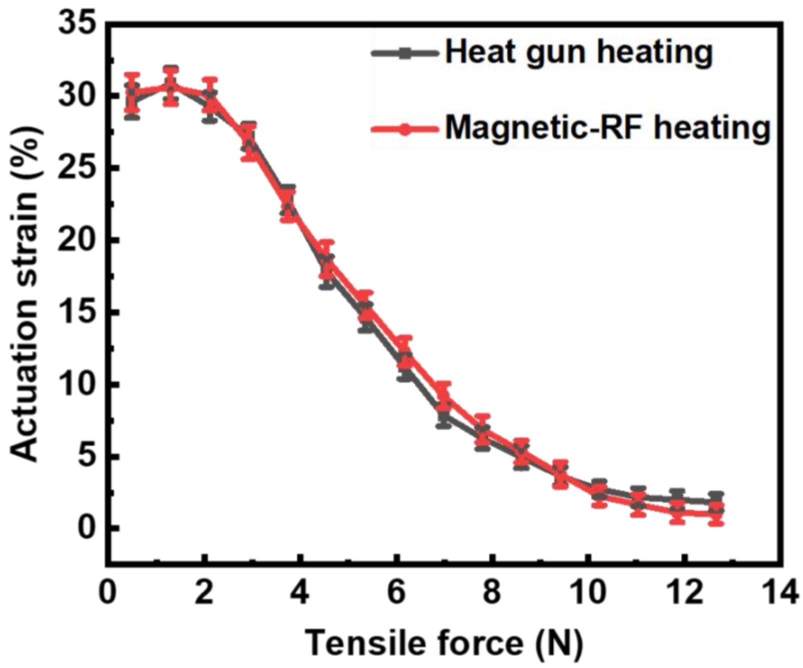

Fig. S17. Measuring the actuation strain with different methods. The comparison of the coiled muscle actuation strain under various loads between the heat gun heating and the RF heating.

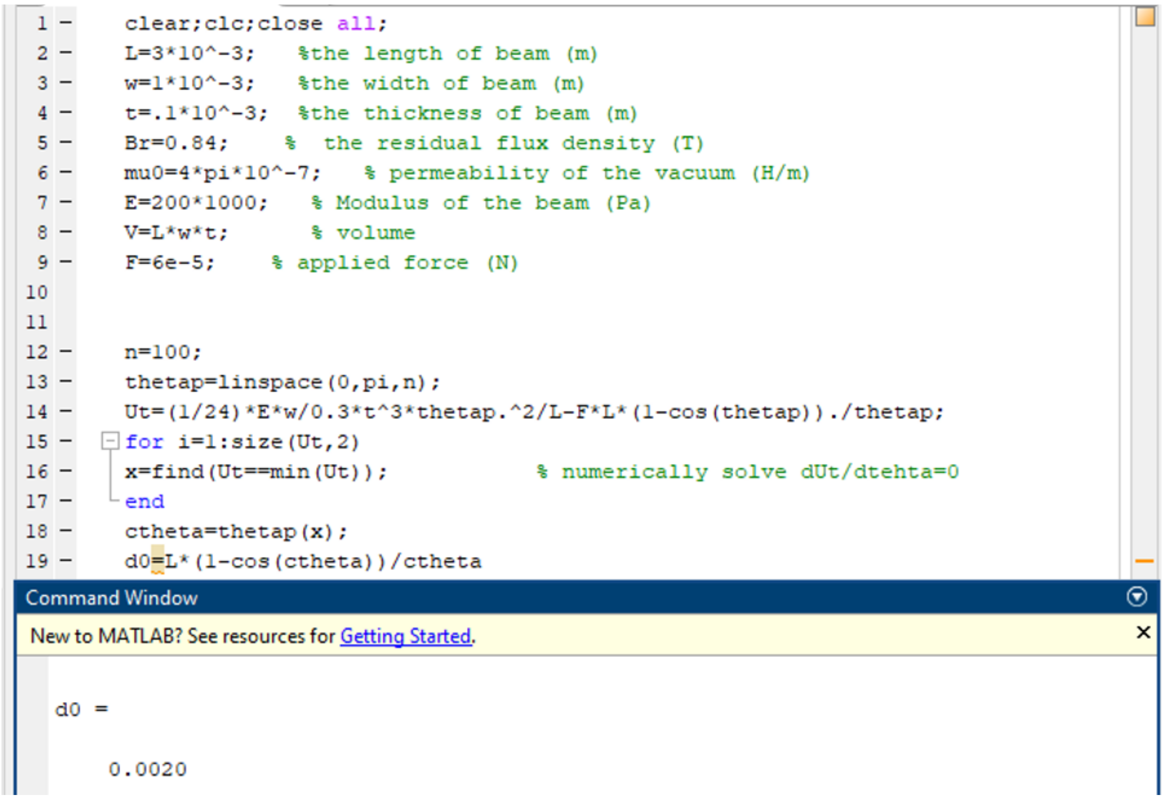

Fig. S18. Output force calculating of the magnetic soft actuator. The Matlab code calculates the output force of the magnetic soft actuator by using the numerical method.

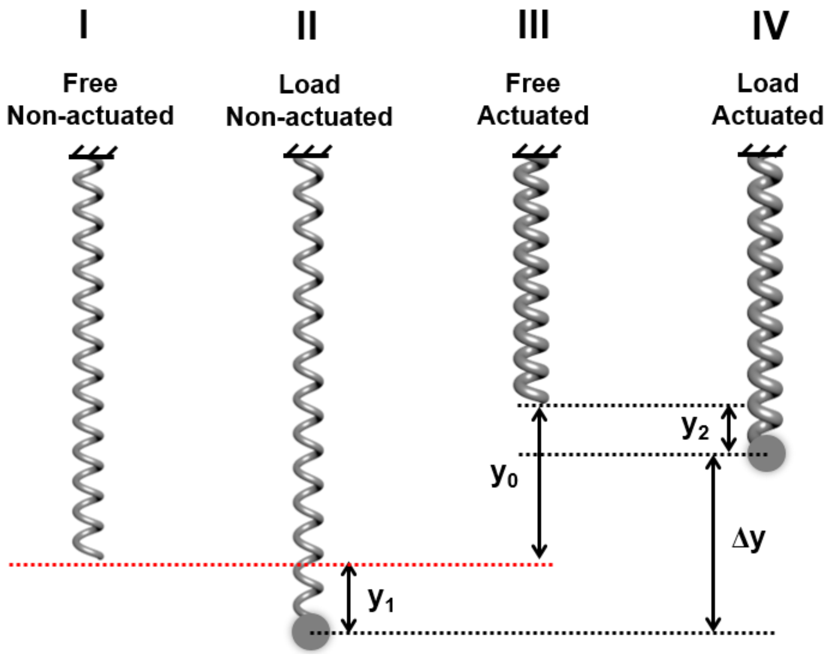

Fig. S19. Calculation of the actuation strain and work capacity of the coiled muscle. Schematic indicating the dependence of length on loading force for actuated and non-actuated coiled muscle.

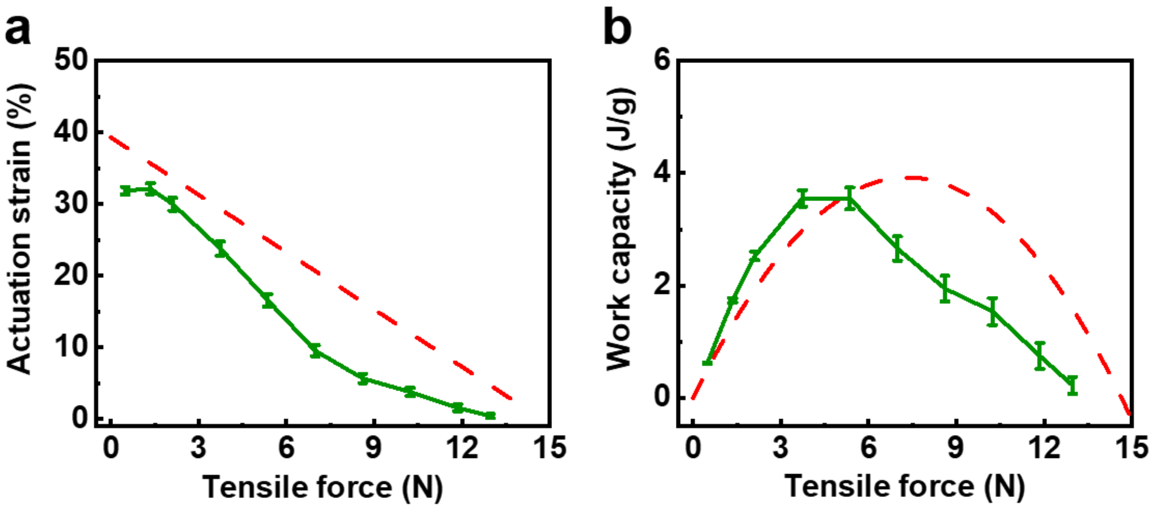

Fig. S20. Comparison of theoretical and experimental results for the coiled muscle. The comparison of (a) actuation strain and (b) work capacity results between the theoretical calculated (red dash line) and experimental measured (green solid line) under different loading forces. Note that the experimental results come from the coiled muscle composed of 4 wt% GO and 200 μm active sheath thickness.

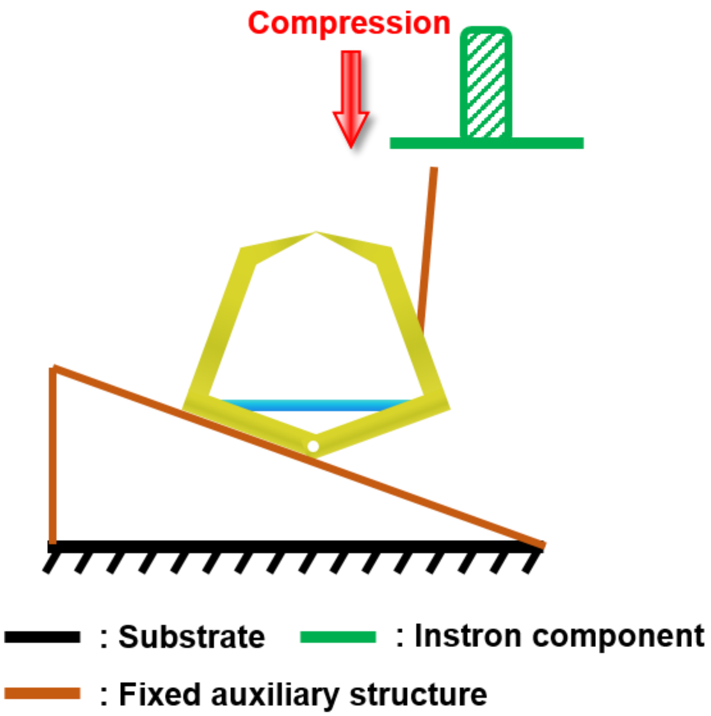

Fig. S21. Measurement schematic for the clamper device. The setup schematic to test the clamping force of the bistable clamper.

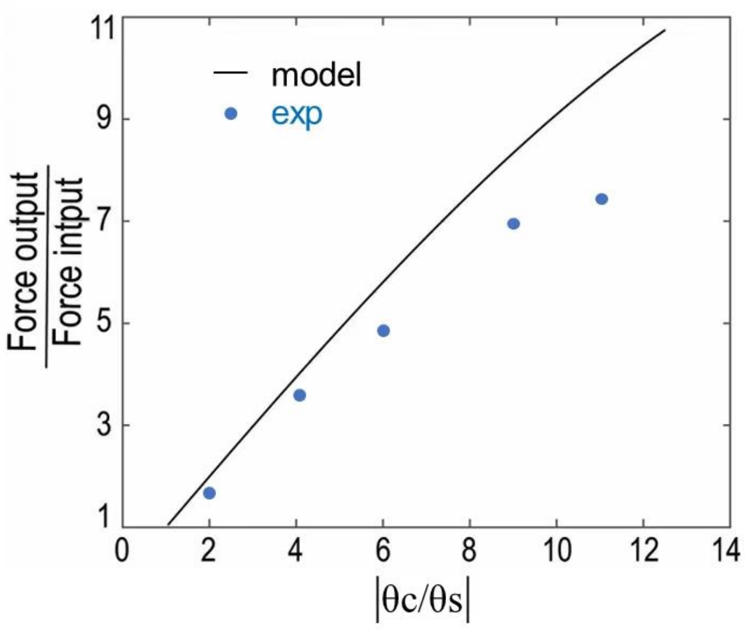

Fig. S22. Optimization of the bistable design. The ratio between output force and input force as a function of θ_c/θ_s.

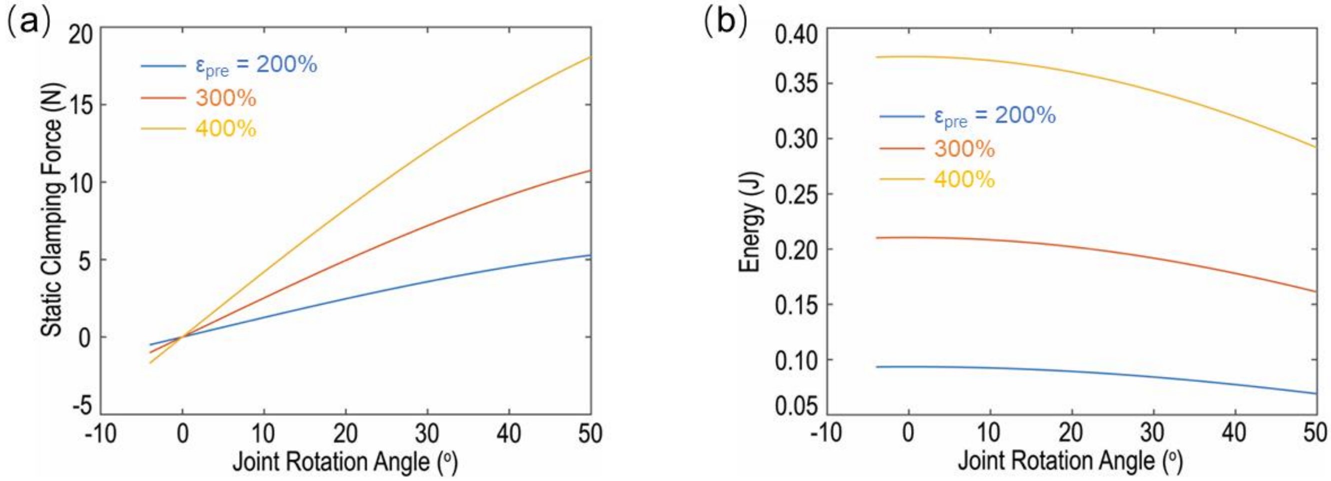

Fig. S23. Static clamping force and potential energy of the bistable clamper. (a) Theoretical static clamping force vs. joint rotation angle for different pre-stretched strains in the elastomer. (b) Theoretical potential energy vs. joint rotation angle for different pre-stretched strains in the elastomer.

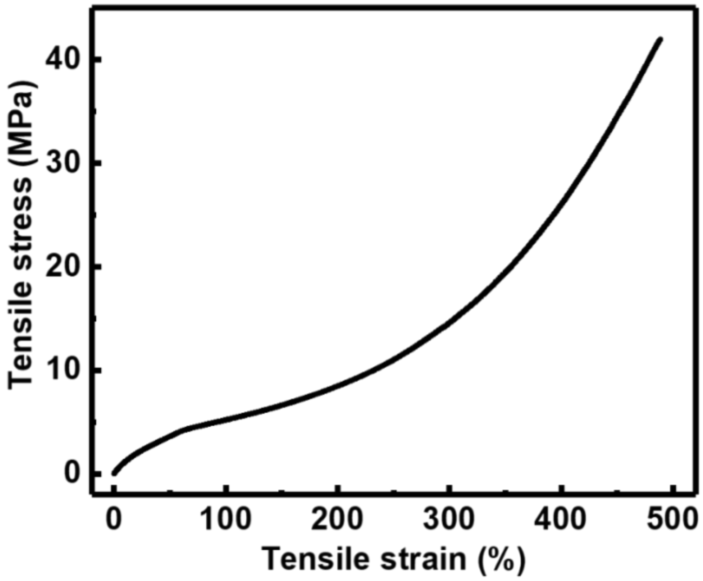

Fig. S24. Characterization of pre-stretched elastomer. The tensile stress-strain curve of the elastomer used for the bistable clamper.

Supplementary Movies

Movie S1. The contractile actuation of the coiled muscle under RF heating.

Movie S2. The torsional behavior of the coiled muscle upon RF heating.

Movie S3. The clockwise rotation and counter-clockwise rotation behavior of the coiled muscle with two sections when actuating the upper and lower section, respectively.

Movie S4. The operation process of the coiled muscle was observed under a microscope camera.

Movie S5. The whole suturing process of the coiled muscle, including the wrapping under magnetic field and the contraction actuated by RF heating.

Movie S6. The cutting behavior of the designed scissor robot under RF heating.

Movie S7. The process of a scissor device to cut the agarose gel pillar off.

Movie S8. The designed driller robot is utilized to drill into the agarose gel.

Movie S9. The clamping behavior of the designed clamper device, which was recorded by the high-speed camera.

Movie S10. The clamper clips the wound on the chicken skin recorded by the high-speed camera.

Movie S11. The walking behavior of the multi-linked coiled muscle under magnetic torque and the contractile actuation under RF heating.

Movie S12. The forward and backward walking of the multi-linked coiled muscle under external magnetic field control.
